# Hypothalamic P62 (SQSTM1) regulates energy balance by modulating leptin signaling

**DOI:** 10.7150/thno.96480

**Published:** 2024-10-07

**Authors:** Shan Yang, Yang Li, Mingyuan Tian, Wuquan Deng, Dongfang Liu, Chen Chen, Zhiming Zhu, Hongting Zheng, Gangyi Yang, Ling Li, Mengliu Yang

**Affiliations:** 1 Department of Endocrinology, the Second Affiliated Hospital, Chongqing Medical University, Chongqing, China.; 2 Department of Clinical Biochemistry and the Key Laboratory of Laboratory Medical Diagnostics in the Ministry of Education, Chongqing Medical University, Chongqing, China.; 3 Department of Endocrinology and Metabolism, Chongqing University Central Hospital, Chongqing Emergency Medical Center, Jiankang Road, Yuzhong District, Chongqing, China.; 4 Endocrinology, SBMS, Faculty of Medicine, University of Queensland, Brisbane, 4072, Australia.; 5 Department of Hypertension and Endocrinology, Daping Hospital, Third Military Medical University, Chongqing Institute of Hypertension, Chongqing, China.; 6 Department of Endocrinology, Xinqiao Hospital, Third Military Medical University, Chongqing, China.

**Keywords:** p62 (SQSTM1), POMC, Obesity, Metabolic disorder, Leptin sensitivity

## Abstract

Rationale: The multifaceted functions of p62 (SQSTM1) are increasingly recognized, but its role in hypothalamic metabolism-associated neurons for energy balance has yet to be elucidated.

Methods: Single-nucleus RNA sequencing (snRNA-Seq) was performed on hypothalamic tissues from db/db and db/m mice to explore p62 expression. Overexpression and knockout of p62 in hypothalamic POMC neurons were performed* via* AAV-mediated gene delivery and Cre-loxP systems. Metabolic outcomes were assessed under normal chow (NCD) and high-fat diet (HFD) conditions. The co-immunoprecipitation and luciferase reporter assays were used to investigate the interaction between p62 and STAT3.

Results: The snRNA-Seq analysis found that p62 was ubiquitously expressed in hypothalamic neurons, with significantly higher levels in POMC neurons of db/db mice compared to db/m controls. Under NCD or HFD conditions, the absence of p62 in POMC neurons led to increased body weight, decreased energy expenditure and leptin sensitivity, while its overexpression in POMC neurons produced the opposite phenotype. Mechanistically, p62 interacts with STAT3, facilitating its phosphorylation to initiate POMC transcription and amplify leptin sensitivity.

Conclusion: This study demonstrated the capacity of p62 to monogenically regulate the obesity phenotype and emphasized its dual role in managing energy homeostasis through direct modulation of STAT3/POMC signaling and amplification of leptin sensitivity.

## Introduction

Obesity is a pivotal contributor to the onset and progression of metabolic diseases, and is a pressing concern in global health 1, 2. Fundamental to obesity is a disruption in energy homeostasis, orchestrated primarily through an imbalance in energy intake and expenditure 3. Under the regulation of the central nervous system (CNS), the intricacies of energy metabolism undergo modulation to maintain physiological homeostasis 4. Hypothalamic nuclei, especially the arcuate nucleus (ARC), play important roles in regulating energy metabolism *in vivo*. The ARC and ventromedial hypothalamus (VMH) house a sophisticated network of neurons that includes synergistic agouti-related protein (AgRP)/neuropeptide Y (NPY) orexigenic neurons and anorexic neurons expressing proopiomelanocortin (POMC), which work in concert to sustain energy balance 5-8. These neurons receive metabolic signals from the periphery and regulate whole-body energy homeostasis 9, 10. Therefore, any genetic (gene expression change) or environmental factor (dietary) can lead to the dysfunction of these neurons, resulting in an imbalance in energy metabolism.

Within this regulatory architecture, the cytoplasmic protein p62 (SQSTM1) has emerged as a potentially significant player, albeit inadequately understudied in the context of the CNS 11, 12. As an important mediator that maintains cell function, p62 is widely expressed in various tissues 13, 14, and is implicated in many metabolic diseases, including adult-onset obesity, nonalcoholic fatty liver disease (NAFLD), and type 2 diabetes (T2D). Previous studies revealed that p62 is involved in some pivotal metabolic processes, such as inflammation and oxidative stress 15, 16, and recent studies have elucidated its involvement in glucose and lipid metabolism *in vivo*, such as adipogenesis 17, insulin signaling 18, adipose tissue browning, and thermogenesis 19. One study reported that whole-brain p62 deficiency leads to hyperphagia, leptin resistance and adult-onset obesity 20. Despite the highlighted significance of p62 in obesity, it is important to recognize that different neurons within the CNS exhibit distinct functions, which may contribute to diverse metabolic outcomes 21. Therefore, the deletion of p62 in the whole-brain does not accurately locate the neurons affected by p62. To describe the exact role of p62 in the CNS, a detailed understanding of its function in different neurons, especially in the hypothalamus, is necessary.

The current study endeavors to unravel the role of p62 in metabolism-sensing neurons in the hypothalamus, aiming to deepen our understanding of its influence on systemic energy homeostasis.

## Results

### Single-nucleus RNA sequencing (snRNA-Seq) analysis of the hypothalamus of mice

**Figure 1:**
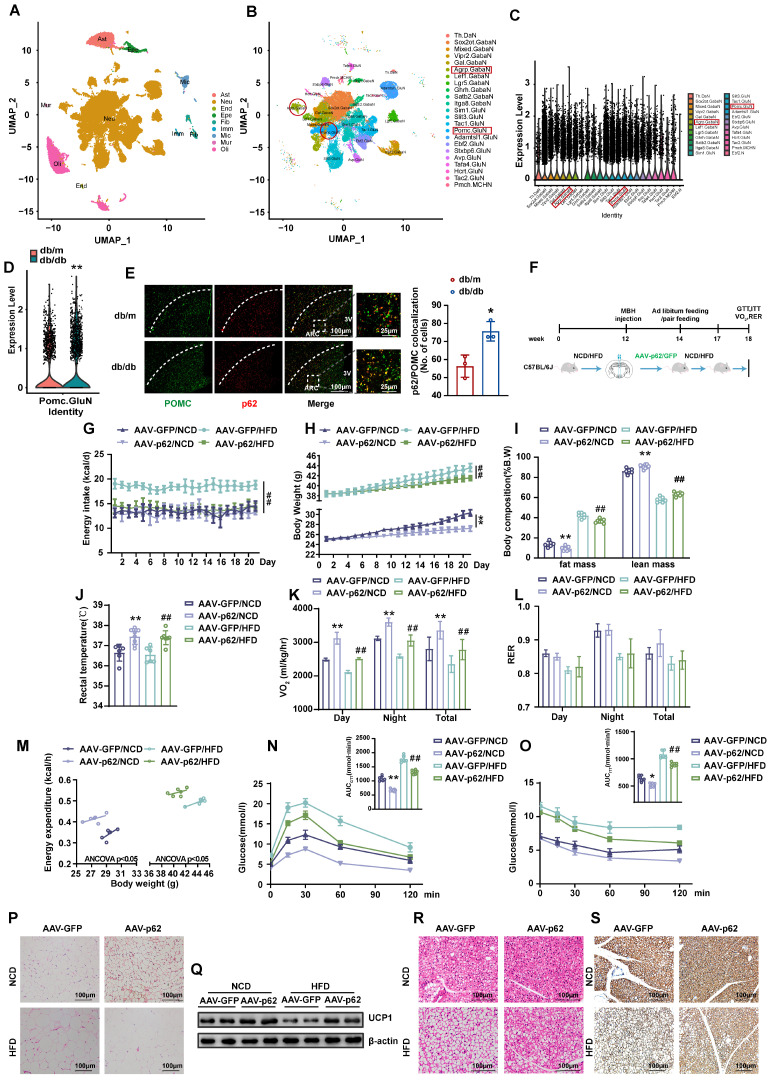
**Overexpression of p62 in the hypothalamus increases energy expenditure and ameliorates systemic metabolism. (A)** UMAP plot showing the clustering of the different cells in the hypothalamus of mice based on snRNA-seq data (n = 3 mice). **(B)** UMAP plot showing the clustering of the different neurons in the hypothalamus of mice based on snRNA-seq data (n = 3 mice). **(C)** Violin plot of p62 expression in hypothalamic neurons based on snRNA-seq data (n= 3 mice).** (D)** Expression of p62 in POMC neurons of db/m and db/db mice (n = 3 mice). **(E)** IF staining of POMC and p62 in the ARC of db/db and db/m mice (n = 3 mice). **(F)** Schematic representation of the experimental procedure. Eight-week-old male WT mice were fed an NCD or HFD for 12 weeks, followed by bilateral MBH injection of AAV9-*p62/GFP* as indicated in the Methods section, and were then maintained on an NCD or HFD for an additional 6 weeks. **(G)** Daily energy intake (n = 6-8 mice). **(H)** Body weight (n = 6-8 mice). **(I)** Body composition (% B.W) (n = 6-8 mice). **(J)** Rectal temperature (n = 6-8 mice). **(K)** 24-h oxygen consumption (V_O2_) (n = 6-8 mice). **(L)** Respiratory exchange ratio (RER: V_CO2_/V_O2_) (n = 6-8 mice). **(M)** ANCOVA of the total energy expenditure versus body weight (n = 6 mice). **(N)** Blood glucose and AUC during the GTT (n = 6-8 mice). **(O)** Blood glucose and AUC during the ITT (n = 6-8 mice). **(P)** H&E staining of WAT (n = 3 mice). **(Q)** UCP1 protein expression in WAT (n = 3 mice). **(R)** H&E staining of BAT (n = 3 mice). **(S)** UCP1 immunostaining in BAT (n = 3 mice). NCD, normal chow diet; HFD, high-fat diet; MBH, mediobasal hypothalamus; ARC, arcuate nucleus; VMH, ventromedial hypothalamus; 3V, third cerebral ventricle; GTT, glucose tolerance test; ITT, insulin tolerance test; AUC, the area under the curve; WAT, white adipose tissue; BAT, brown adipose tissue. Data are expressed as the mean ± SD. *p < 0.05, **p < 0.01 *vs.* GFP/NCD or db/m;^ ##^ *p* < 0.01* vs.* GFP/HFD.

To delineate the expression pattern of p62 in the CNS, particularly in hypothalamic neurons, we performed a snRNA-seq analysis on hypothalamic tissues from db/db mice, using db/m mice as controls. The present investigation revealed the ubiquitous presence of p62 across most neurons, including those pivotal to metabolism, such as POMC and AgRP neurons (Fig. 1A - C and Fig. S1A - E). Notably, a significant difference in p62 expression was observed in POMC, but not AgRP neurons between db/db and db/m mice, highlighting the potential involvement of p62 in metabolic processes (Fig. 1D and Fig. S1F). To substantiate the snRNA-Seq results, we utilized immunohistochemical (IHC) staining, which confirmed the pronounced expression of p62 in pivotal metabolic regions, the hypothalamic ARC and VMH (Fig. S2A). Furthermore, immunofluorescence (IF) staining also showed that p62 expression in POMC neurons was greater in db/db mice than in db/m mice (Fig. 1E), which is consistent with our snRNA-seq results. These findings further underscore the prospective central role of p62 in modulating energy metabolism.

### Overexpression of p62 in the hypothalamus promotes white adipose tissue (WAT) browning and increases energy expenditure

To understand the effect of hypothalamic p62 on systemic energy balance, we bilaterally injected AAV9-*p62/GFP* into the mediobasal hypothalamus (MBH) of normal chow diet (NCD)- or high-fat diet (HFD)-fed WT mice to overexpress p62 in the hypothalamus (Fig. 1F). The localization and efficacy of the stereotactic injection in the hypothalamus were confirmed by IF staining for GFP and p62 (Fig. S2B). Notably, relative to their AAV9-*GFP* counterparts, mice subjected to a HFD and infected with AAV9-*p62* exhibited a substantial reduction in energy intake (Fig. 1G), while NCD-fed mice showed no change in energy intake. Remarkably, irrespective of diet (NCD or HFD), a significant reduction in body weight (BW) was consistently noted (Fig. 1H). The decreased weight could be attributed to reductions in adiposity coupled with an increase in lean mass (Fig. 1I). Moreover, the increase in rectal temperature (Fig. 1J) suggested an increase in thermogenesis in the AAV9-*p62-*injected mice. This observation paralleled our pair-feeding experiment results (Fig. S2C and D), implying that p62 may modulate weight gain by amplifying peripheral energy expenditure under normal conditions without affecting energy intake. In contrast, in metabolic disorder models, p62 appears to decelerate obesity by simultaneously inhibiting energy intake and enhancing energy expenditure. Metabolic cage analysis further corroborated these findings, demonstrating elevated total energy expenditure (24-h O_2_ consumption, V_O2_) in the AAV9-*p62*-infected mice, regardless of diet type. The respiratory exchange ratio (RER) was unchanged (Fig. 1K and L). Furthermore, AAV9-*p62*-infected mice exhibited increased energy expenditure in relation to BW (Fig. 1M), suggesting an increase in overall energy dynamics in AAV9-*p62*-injected mice.

Additionally, both diet groups, when subjected to hypothalamic MBH injection of AAV9-*p62*, showed improvements in glucose regulation, as evidenced by glucose tolerance test (GTT) and insulin tolerance test (ITT) (Fig. 1N and O). Consistent with the above metabolic phenotypes, the decreased adiposity in hypothalamic p62-overexpressing mice was accompanied by elevated UCP-1 protein expression (Fig. 1P and Q). Similar changes were also observed in brown adipose tissue (BAT) (Fig. 1R and S). These results demonstrated that central p62 promotes WAT browning and thermogenesis. Taken together, these observations suggest that in diverse metabolic contexts, p62 might exert multifaceted effects on managing energy metabolism.

### Overexpression of p62 in the hypothalamus upregulates POMC expression and STAT3 signaling in both NCD- and HFD-fed mice

**Figure 2:**
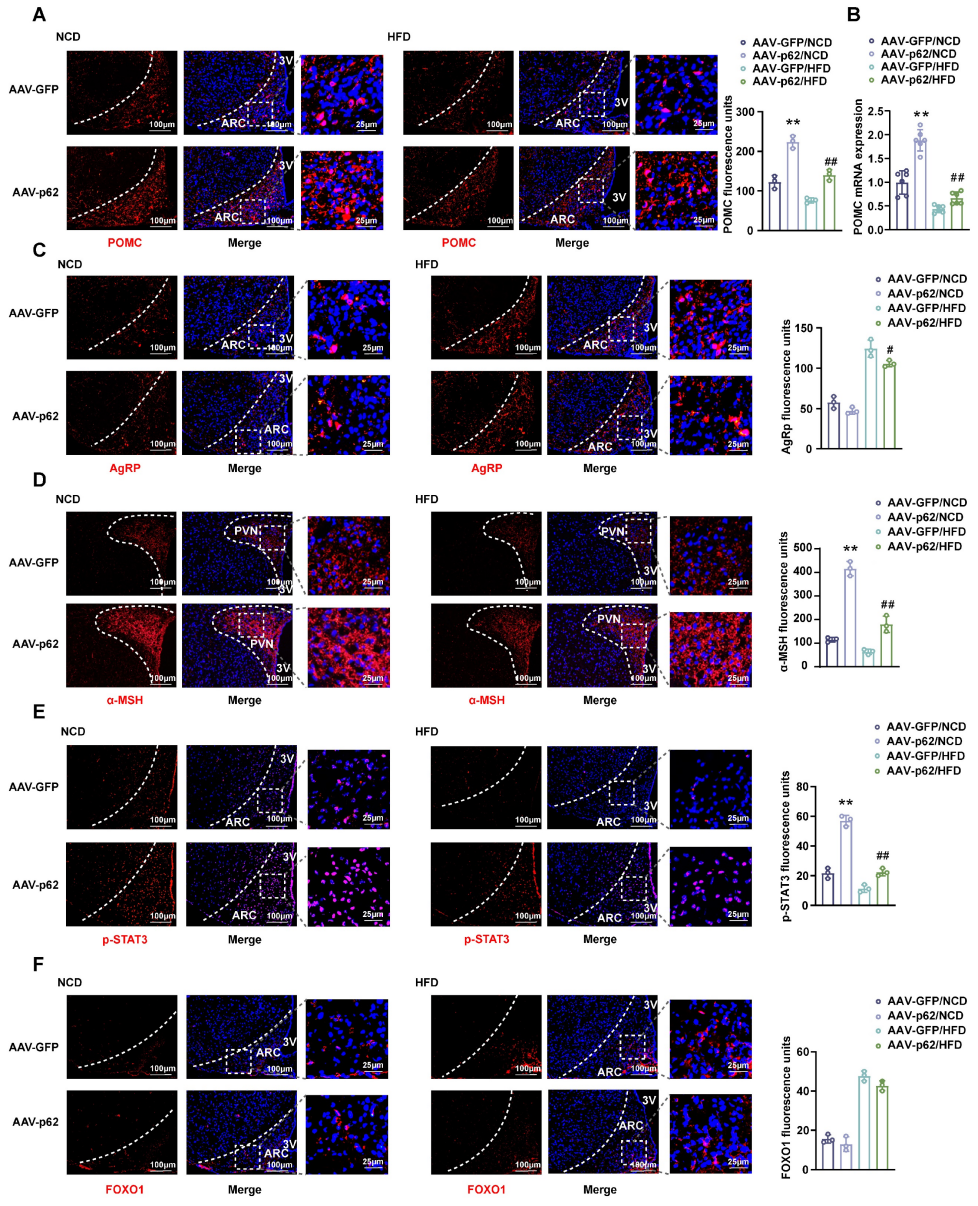
** Effect of p62 overexpression in the hypothalamus on the expression of POMC and AgRP and on leptin signaling.** Eight-week-old male WT mice were fed an NCD or HFD for 12 weeks, followed by bilateral MBH injection of AAV9-*p62/GFP*, and were then maintained on an NCD or HFD for an additional 6 weeks. Brain sections were cut and collected as indicated in the Methods section. **(A)** IF staining for POMC (left) and RFU (right) in the ARC (n = 3 mice). **(B)** POMC mRNA expression in the hypothalamus (n = 6 mice). **(C)** IF staining for AgRP (left) and RFU (right) in the ARC (n = 3 mice). **(D)** IF staining for α-MSH (left) and RFU (right) in the PVN (n = 3 mice). **(E)** IF staining for STAT3 phosphorylation (left) and RFU (right) in the ARC (n = 3 mice). **(F)** IF staining for FOXO1 (left) and RFU (right) in the ARC (n = 3 mice). NCD, normal chow diet; HFD, high-fat diet; MBH, mediobasal hypothalamus; ARC, arcuate nucleus; PVN, paraventricular nucleus; 3V, third cerebral ventricle; RFU, relative fluorescent units. Data are expressed as the mean ± SD. *p < 0.05, **p < 0.01 *vs.* GFP/NCD group; ^##^*p* < 0.01 *vs*. GFP/HFD group.

Considering the pivotal roles of POMC and AgRP neurons in energy homeostasis within the hypothalamic ARC 22, we assessed the effect of hypothalamic p62 overexpression on these neurons. IF images and mRNA expression analysis revealed an upregulated POMC expression in both NCD- and HFD-fed mice infected with AAV9-*p62* compared to their AAV9-*GFP* counterparts (Fig. 2A and B). However, only HFD-fed AAV9-*p62* mice exhibited decreased AgRP expression (Fig. 2C). In addition, p62 overexpression amplified the levels of α-melanocyte stimulating hormone (α-MSH), a bioactive neuropeptide generated by POMC, under both dietary regimens (Fig. 2D), underscoring the potential of hypothalamic p62 in modulating POMC expression and α-MSH release.

Given the regulatory dynamics of leptin and insulin on energy metabolism and BW through POMC and AgRP neurons in the hypothalamus, which are orchestrated through pathways such as JAK2/STAT3 and Akt/FOXO1 23, our investigation expanded to assess the effects of hypothalamic p62 overexpression on these specific pathways*.* Remarkably, phosphorylated STAT3 (p-STAT3) levels within the ARC of AAV9-*p62* mice were increased under both dietary conditions compared to controls, while FOXO1 levels remained unchanged (Fig. 2E and F). Collectively, these results suggest a potential role of hypothalamic p62 signaling in manipulating POMC expression and STAT3 phosphorylation, thereby regulating systemic metabolism.

### p62 re-expression in hypothalamus increases energy expenditure and facilitates leptin signaling in p62 global knockout (p62^-/-^) mice

**Figure 3:**
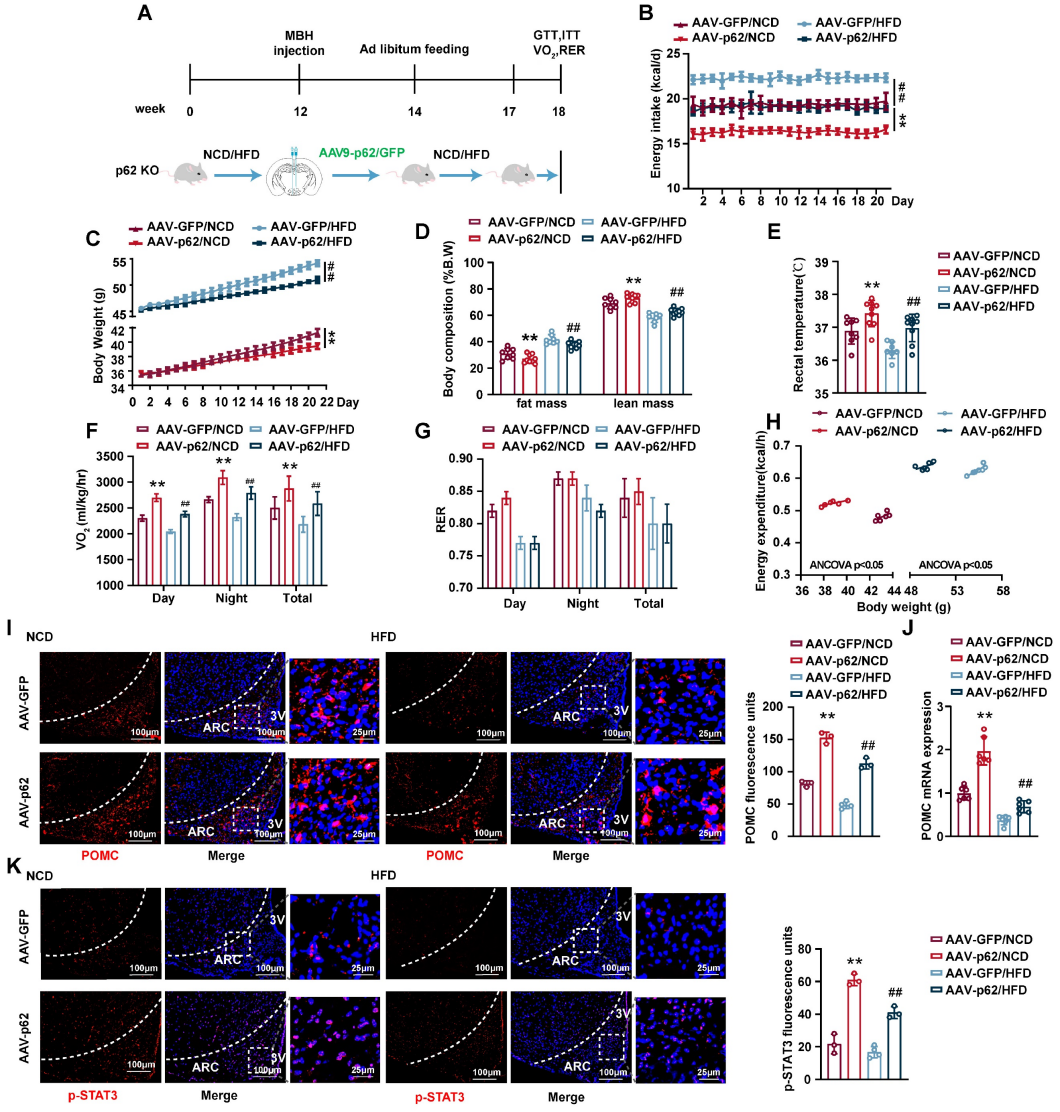
** p62 re-expression in the hypothalamus increases energy expenditure and promotes leptin signaling in p62^-/-^ mice. (A)** Schematic representation of the experimental procedure. Eight-week-old male p62^-/-^ mice were fed an NCD or HFD for 12 weeks, followed by bilateral MBH injection of AVV9-*p62/GFP* as indicated in the Methods section, and were then maintained on an NCD or HFD for an additional 6 weeks. **(B)** Energy intake (n = 7-9 mice). **(C)** Body weight (n = 7-9 mice). **(D)** Body composition (% B.W) (n = 7-9 mice). **(E)** Rectal temperature (n = 7-9 mice). **(F)** 24-h oxygen consumption (Vo_2_) (n = 7-9 mice). **(G)** Respiratory exchange ratio (RER: V_CO2_/V_O2_) (n = 7-9 mice). **(H)** ANCOVA of the total energy expenditure versus body weight (n = 6-7 mice). **(I)** IF staining of POMC (left) and RFU (right) in the ARC (n = 3-4 mice). **(J)** POMC mRNA expression in the hypothalamus (n = 6 mice). **(K)** IF staining of STAT3 phosphorylation (left) and RFU (right) in the ARC (n = 3-4 mice). NCD, normal chow diet; HFD, high-fat diet; MBH, mediobasal hypothalamus; 3 V, third cerebral ventricle; ARC, arcuate nucleus; RFU, relative fluorescent units. Data are expressed as the mean ± SD. *p < 0.05, **p < 0.01 *vs.* GFP/NCD; ^##^*p* < 0.01 *vs*. GFP/HFD.

To further substantiate the central regulatory role of p62 in systemic metabolism and to exclude the potential effects of peripheral tissue p62 on the metabolic phenotype, we generated p62^-/-^ mice and administered bilateral AAV9-*p62*/*GFP* to the MBH of p62^-/-^ mice (Fig. 3A). This model recapitulated the anti-obesity outcomes previously noted in MBH AAV9-*p62* WT mice, underscoring the central role of MBH p62 expression in mitigating obesity indicators, including decreased energy intake, BW and fat mass, alongside increased rectal temperature and energy expenditure (Fig. 3B-H), signifying enhanced metabolic health. The beneficial metabolic alterations were mirrored in glucose and lipid regulation under both NCD and HFD regimens (Fig. S3A-F). Furthermore, we observed a marked increase in both POMC expression and STAT3 phosphorylation in the ARC of p62^-/-^ mice post-AVV9-*p62* treatment (Fig. 3I-K), underlining the central role of this pathway in metabolic regulation of p62. Collectively, we further confirmed the regulatory role of central p62 in systemic metabolism.

### Selective overexpression of p62 in POMC neurons increases energy expenditure and facilitates leptin/JAK2/STAT3 signaling

While previous studies have employed non-cell type specific approaches, the intricate mechanism linking cell-specific p62 action to metabolic phenotypes has not been elucidated. Since AgRP and POMC neurons are recognized as “first-order” entities in orchestrating the feeding response to nutritional cues 24, we aimed to explore the metabolic repercussions of p62 overexpression in POMC- and AgRP-expressing neurons*.* Employing a Cre-dependent approach, AAV expressing p62 or GFP (AAV9-DIO-*p62/GFP,* DIO, the double-floxed inverse orientation) was administered to the ARC of POMC-Cre or AgRP-Cre mice to specifically overexpress p62 in POMC (POMC-*p62* OE mice) or AgRP (AgRP-*p62* OE mice).

**Figure 4:**
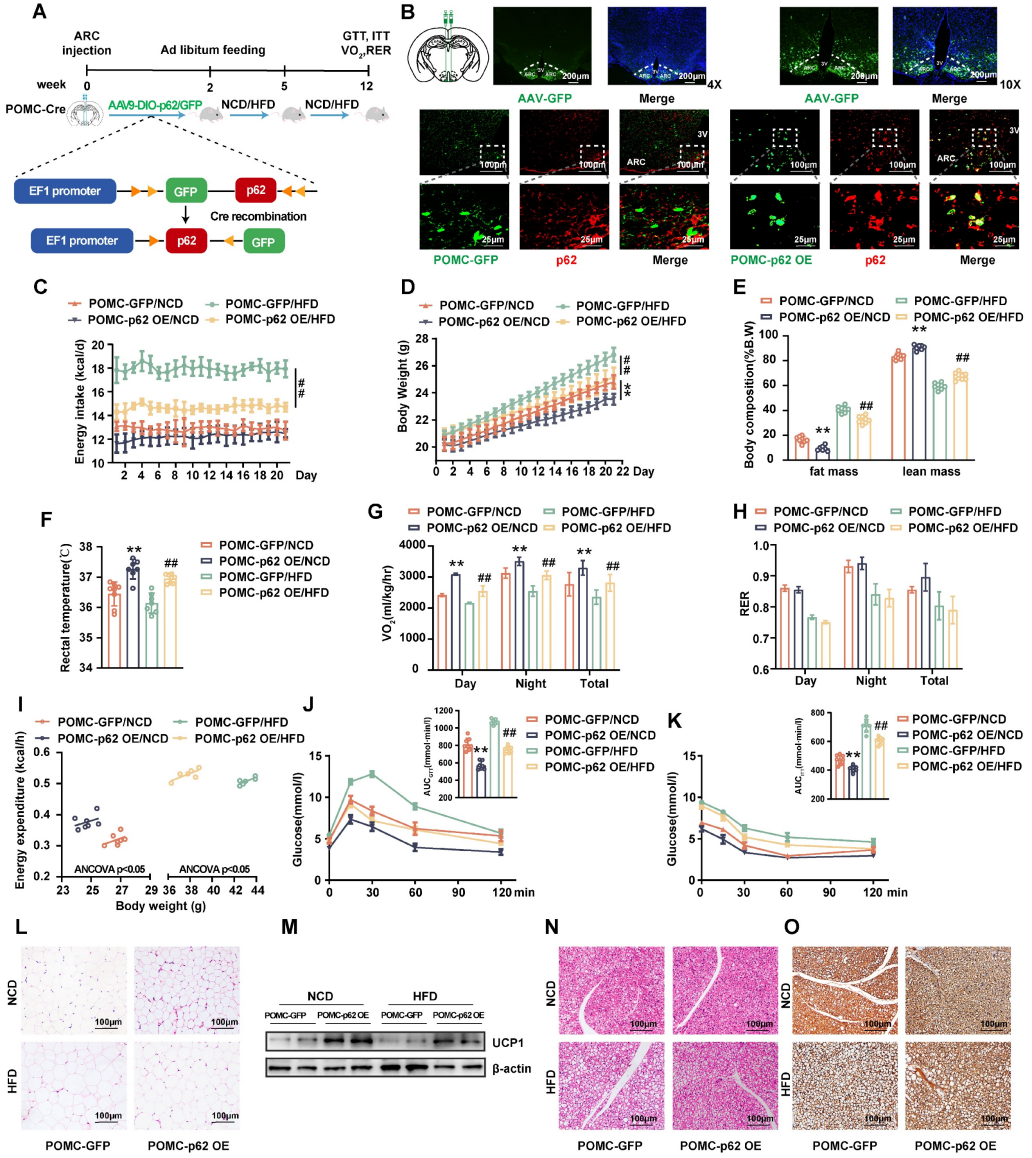
** Selective overexpression of p62 in POMC neurons increases energy expenditure. (A)** Schematic representation of the experimental procedure. Eight-week-old male POMC-Cre mice received a bilateral ARC injection of AVV9-DIO-*p62/GFP* and were fed a NCD or HFD for 12 weeks. **(B)** IF staining for the distribution of AVV9-DIO-*GFP/p62* in the ARC (upper) and p62 expression (lower) in POMC neurons. **(C)** Energy intake (n = 7-8 mice). **(D)** Body weight (n = 7-8 mice). **(E)** Body composition (n = 7-8 mice). **(F)** Rectal temperature (n = 7-8 mice). **(G)** 24-h oxygen consumption (Vo_2_) (n = 7-8 mice). **(H)** Respiratory exchange ratio (RER: V_CO2_/V_O2_) (n = 7-8 mice). **(I)** ANCOVA of the total energy expenditure versus body weight (n = 6-7 mice). **(J** and **K)** Blood glucose and AUC during the GTT (J) and ITT (K) (n = 7-8 mice). **(L)** H&E staining of WAT (n = 3 mice). **(M)** UCP1 protein expression in WAT (n = 3 mice). **(N)** H&E staining of BAT (n = 3 mice). **(O)** UCP1 immunostaining in BAT (n = 3 mice). NCD, normal chow diet; HFD, high-fat diet; GTT, glucose tolerance test; ITT, insulin tolerance test; AUC, the area under the curve; 3 V, third cerebral ventricle; ARC, arcuate nucleus; WAT, white adipose tissue; BAT, brown adipose tissue. Data are expressed as the mean ± SD. ** *p* < 0.01 *vs.* POMC-GFP/NCD; ^##^*p* < 0.01 *vs*. POMC-GFP/HFD.

**Figure 5:**
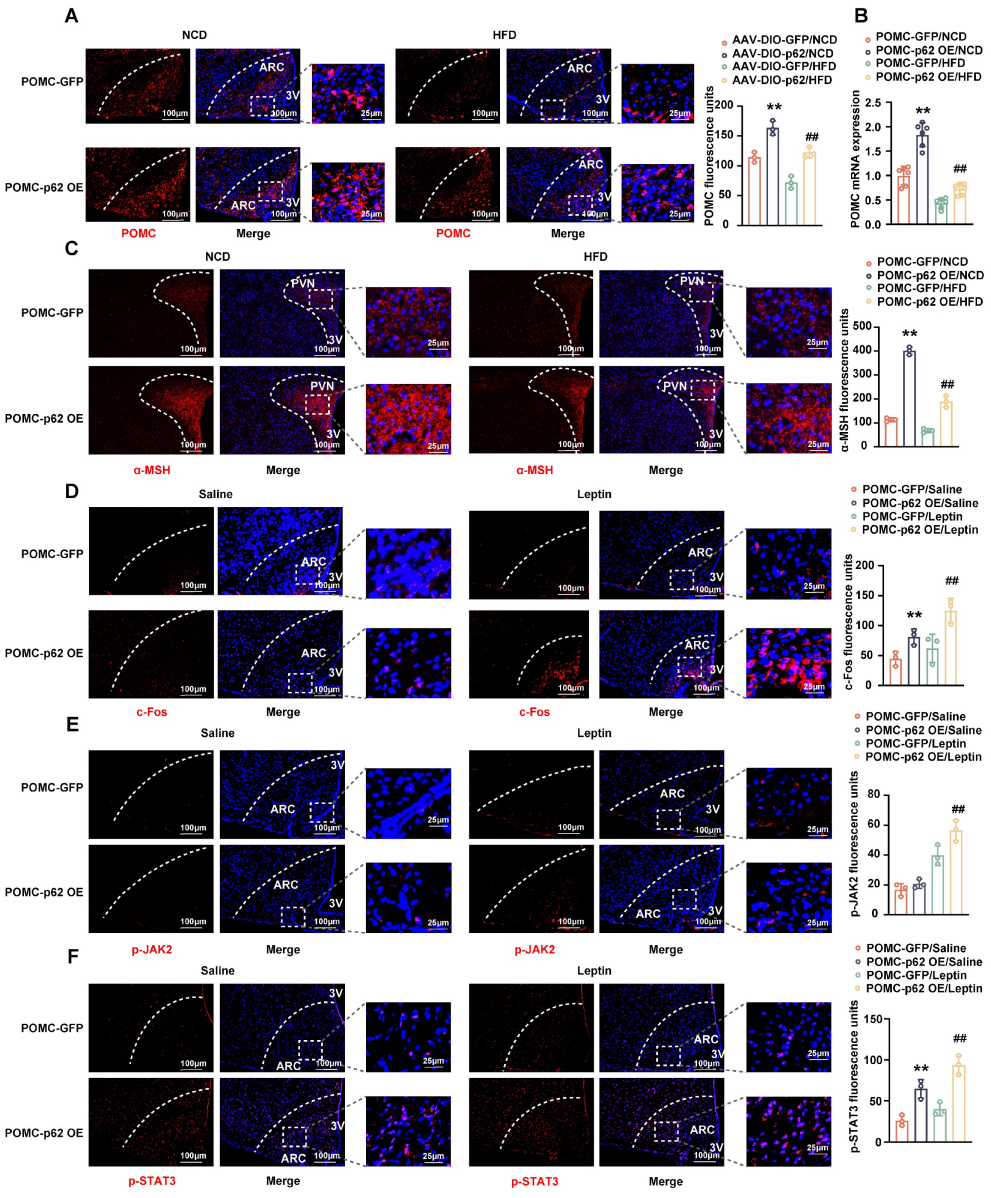
** Effects of p62 overexpression in POMC neurons on JAK2 and STAT3 phosphorylation.** Eight-week-old male POMC-Cre mice received a bilateral ARC injection of AVV9-DIO-*p62/GFP* and were fed a NCD or HFD for 12 weeks. Two subgroups of mice were intraperitoneally injected with leptin/saline twice a day for 3 days, as indicated in the Methods section. **(A)** IF staining of POMC (left) and RFU (right) in the ARC (n = 3 mice). **(B)** POMC mRNA expression in the hypothalamus (n = 6 mice). **(C)** IF staining for α-MSH (left) and RFU (right) in the PVN (n = 3 mice). **(D)** IF staining for c-Fos (left) and RFU (right) in the ARC (n = 3 mice). **(E)** IF staining of phosphorylated JAK2 (left) and RFU (right) in the ARC (n = 3 mice). **(F)** IF staining for phosphorylated STAT3 (left) and RFU (right) in the ARC (n = 3 mice). NCD, normal chow diet; HFD, high-fat diet; 3V, third cerebral ventricle; ARC, arcuate nucleus; PVN, paraventricular nucleus; RFU, relative fluorescent units. Data are expressed as the mean ± SD. **p < 0.01 *vs.* POMC-GFP/NCD or POMC-GFP/saline; ^##^*p* < 0.01 *vs*. POMC-GFP/HFD or POMC-GFP/leptin.

These mice were then subjected to either NCD or HFD for 12 weeks (Fig. 4A and Fig. S4A). IF staining verified effective viral distribution in the hypothalamus (Fig. 4B upper) and a marked upregulation of p62 (red) in POMC neurons (green) of the POMC-*p62* OE mice relative to those of the controls (Fig. 4B, lower). POMC-*p62* OE mice exhibited diminished BW and fat mass alongside elevated rectal temperature and oxygen consumption across diets, with only HFD-fed mice mirroring the reduced energy intake reported in our earlier findings (Fig. 4C-G). Despite these shifts, the RER (V_CO2_/V_O2_) remained unchanged (Fig. 4H). In addition, POMC-*p62*-OE mice showed elevated energy expenditure in relation to BW (Fig. 4I). Further analysis revealed that p62 overexpression in POMC neurons enhanced both glucose tolerance and clearance, and promoted adipose tissue browning and thermogenesis (Fig. 4J-O). Moreover, elevated expression levels of POMC and α-MSH were noted in the hypothalamus irrespective of dietary conditions (Fig. 5A-C).

Transitioning our focus to the intricate interplay between neuronal excitability, p62 and leptin/STAT3 signaling, we administered leptin intraperitoneally to NCD-fed POMC-*p62* OE mice for three days. IF staining revealed an increase in c-FOS and leptin-mediated JAK2 phosphorylation (Fig. 5D and E). Notably, the overexpression of p62 in POMC neurons also significantly increased STAT3 phosphorylation, regardless of leptin stimulation (Fig. 5F), suggesting that p62 enhances both endogenous and exogenous leptin effects. Parallel studies in AgRP-*p62* OE mice revealed similar outcomes pertaining to energy intake, BW, rectal temperatures, and glucose and insulin tolerance, although these findings were constrained to the HFD condition (Fig. S4B-F). Corresponding to these changes, AgRP expression was inhibited in the ARC of HFD-fed AgRP-*p62* OE mice (Fig. S4G). Collectively, these data reveal that p62 acts on POMC or AgRP neurons to promote POMC expression, but inhibits AgRP expression to regulate energy metabolism and prevent obesity.

### Inducible loss of p62 in POMC neurons reduces energy expenditure and exacerbates metabolic dysfunction

Having extensively explored the implications of hypothalamic p62 overexpression, it is now imperative to elucidate the consequences of its knockdown within the same region to furnish a holistic understanding of its function. To mitigate developmental concerns, such as POMC lineage cells maturing into orexigenic AgRP neurons 25, we generated POMC-*p62* KO mice by crossbreeding POMC-Cre^ER^ mice with p62^fl/fl^ mice. After administering tamoxifen at 8 weeks, the mice were subjected to either NCD or HFD for 12 weeks (Fig. S5A). These POMC-*p62* KO mice exhibited diminished p62 expression in POMC neurons (Fig. S5B), increased BW and fat mass, reduced energy expenditure, and impaired glucose and insulin responses in both NCD and HFD mice, while altered energy intake was observed exclusively in the HFD group (Fig. S5C-K). Morphologically, these mice displayed hypertrophic adipocytes coupled with a decrease in Ucp1 expression in both WAT and BAT (Fig. S5L-O). Downregulation of both POMC and α-MSH in the hypothalamus was observed, irrespective of the diet (Fig. S6A-C). Furthermore, p62 knockdown in POMC neurons attenuated leptin-stimulated c-Fos expression within the ARC (Fig. S6D), concomitant with reduced leptin-induced JAK2 phosphorylation (Fig. S6E). Additionally, a decline in STAT3 phosphorylation was observed, regardless of the presence of leptin cues (Fig. S6F). Taken together, these findings establish that p62 modulates POMC neurons preferentially through leptin-dependent pathways.

### Deletion of p62 in ObRb-expressing neurons results in a decrease in energy expenditure and a deterioration of metabolic disorders

**Figure 6:**
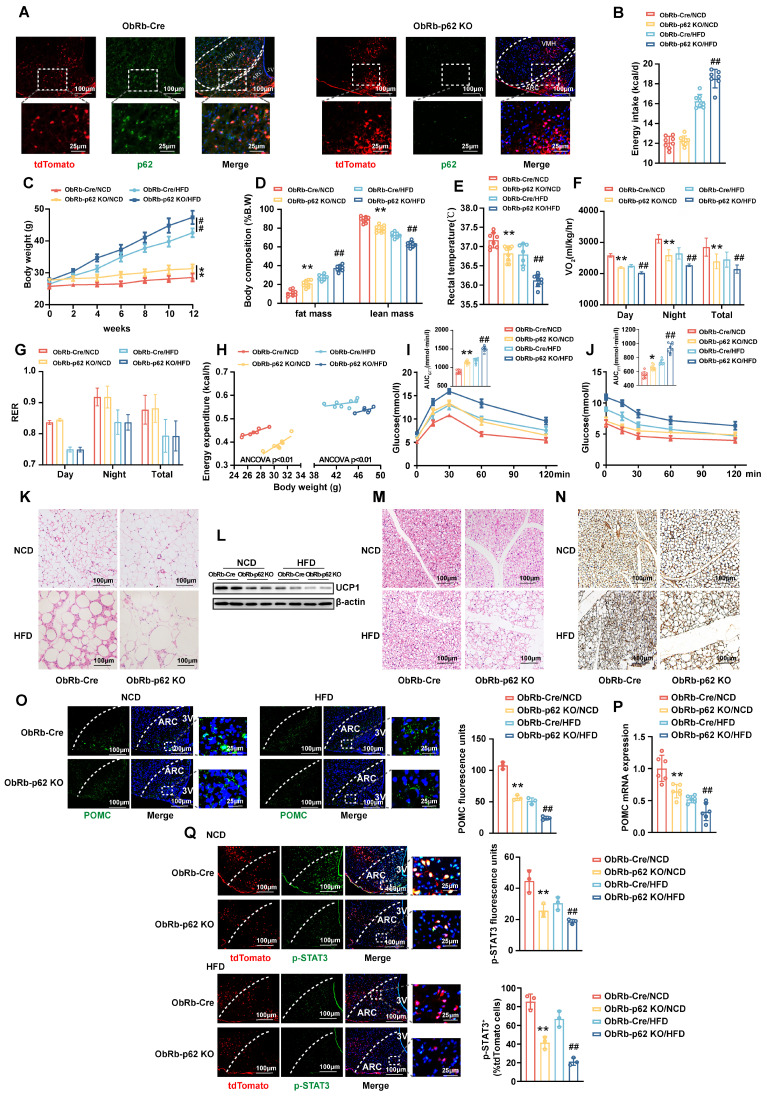
**Deletion of p62 in ObRb-expressing neurons results in a decrease in energy expenditure and a deterioration of metabolic disorders in both lean and obese mice.** The p62^flox/flox^ mice were crossed with ObRb-*Cre*:tdTomato mice to generate ObRb-p62 KO mice with tdTomato. Eight-week-old male ObRb-p62 KO and ObRb-*Cre*:tdTomato mice were fed a NCD or HFD for 12 weeks, as indicated in the Methods section. **(A)** IF staining of ObRb neurons with tdTomato (red) and p62 (green) in the ARC and VMH. **(B)** Average energy intake (n = 7-8 mice). **(C)** Body weight (n = 7-9 mice). **(D)** Body composition (n = 7-9 mice). **(E)** Rectal temperature (n = 7-9 mice). **(F)** 24-h oxygen consumption (Vo_2_) (n = 7-9 mice). **(G)** Respiratory exchange ratio (RER: V_CO2_/V_O2_) (n = 7-9mice). **(H)** ANCOVA of the total energy expenditure versus body weight (n = 6-8 mice). **(I)** Blood glucose and AUC during the GTT (n = 7-9 mice). **(J)** Blood glucose and AUC during the ITT (n = 7-9 mice). **(K)** H&E staining of WAT (n = 3 mice).** (L)** UCP1 protein expression in WAT (n = 3 mice). **(M)** H&E staining of BAT (n = 3 mice). **(N)** UCP1 immunostaining in BAT (n = 3 mice). **(O)** IF staining for POMC expression (left) and RFU (right) in the ARC (n = 3 mice). **(P)** POMC mRNA expression in the hypothalamus (n = 6 mice). **(Q)** IF staining for phosphorylated STAT3 (left) and RFU (right) in ObRb neurons (n = 3mice). 3V, third cerebral ventricle; ARC, arcuate nucleus. VMH, ventromedial hypothalamus; NCD, normal chow diet; HFD, high-fat diet; GTT, glucose tolerance test; ITT, insulin tolerance test; AUC, the area under the curve; WAT, white adipose tissue; BAT, brown adipose tissue; RFU, relative fluorescent units. Data are expressed as the mean ± SD. *p < 0.05, **p < 0.01 *vs.* ObRb-Cre/NCD; ^##^ *p* < 0.01 *vs*. ObRb-Cre/HFD.

The JAK2/STAT3 signaling axis emerges as an integral response to leptin receptor (ObRb) engagement, orchestrating the dynamic expression of POMC and AgRP. Notably, under physiological conditions, leptin is posited to primarily activate the JAK2/STAT3 pathway within the ARC 23. To further elucidate the interplay between p62 and leptin signaling, we generated ObRb-*p62* KO mice carrying tdTomato by crossing p62^fl/fl^ mice with ObRb*-Cre* mice, which express the tdTomato fluorescent protein in ObRb-expressing neurons. Eight-week-old male ObRb-*p62* KO and ObRb-*Cre* mice were fed either a NCD or HFD for 12 weeks (Fig. S7A). A marked reduction in p62 expression was observed in the ARC of ObRb-*p62* KO mice compared to ObRb-*Cre* mice (Fig. 6A). This deletion resulted in significant physiological alterations, delineated by reduced energy expenditure and exacerbated metabolic disorders, especially under HFD feeding (Fig. 6B-N). Furthermore, the deletion of p62 in ObRb-expressing neurons significantly inhibited POMC and α-MSH expression in the ARC and PVN (Fig. 6O and P; Fig. S7B), respectively, and suppressed STAT3 phosphorylation (Fig. 6Q) under both dietary conditions. In contrast, AgRP expression was increased only under HFD feeding (Fig. S7C), with FOXO1 remaining unchanged (Fig. S7D). Taken together, these data highlight the crucial role of p62 within ObRb neurons, highlighting its influence on metabolic homeostasis through the modulation of leptin-ObRb/STAT3 signaling.

### Overexpression of p62 in the MBH of ob/ob mice alleviates obesity induced by leptin deficiency and promotes the anti-obesity effect of leptin

**Figure 7:**
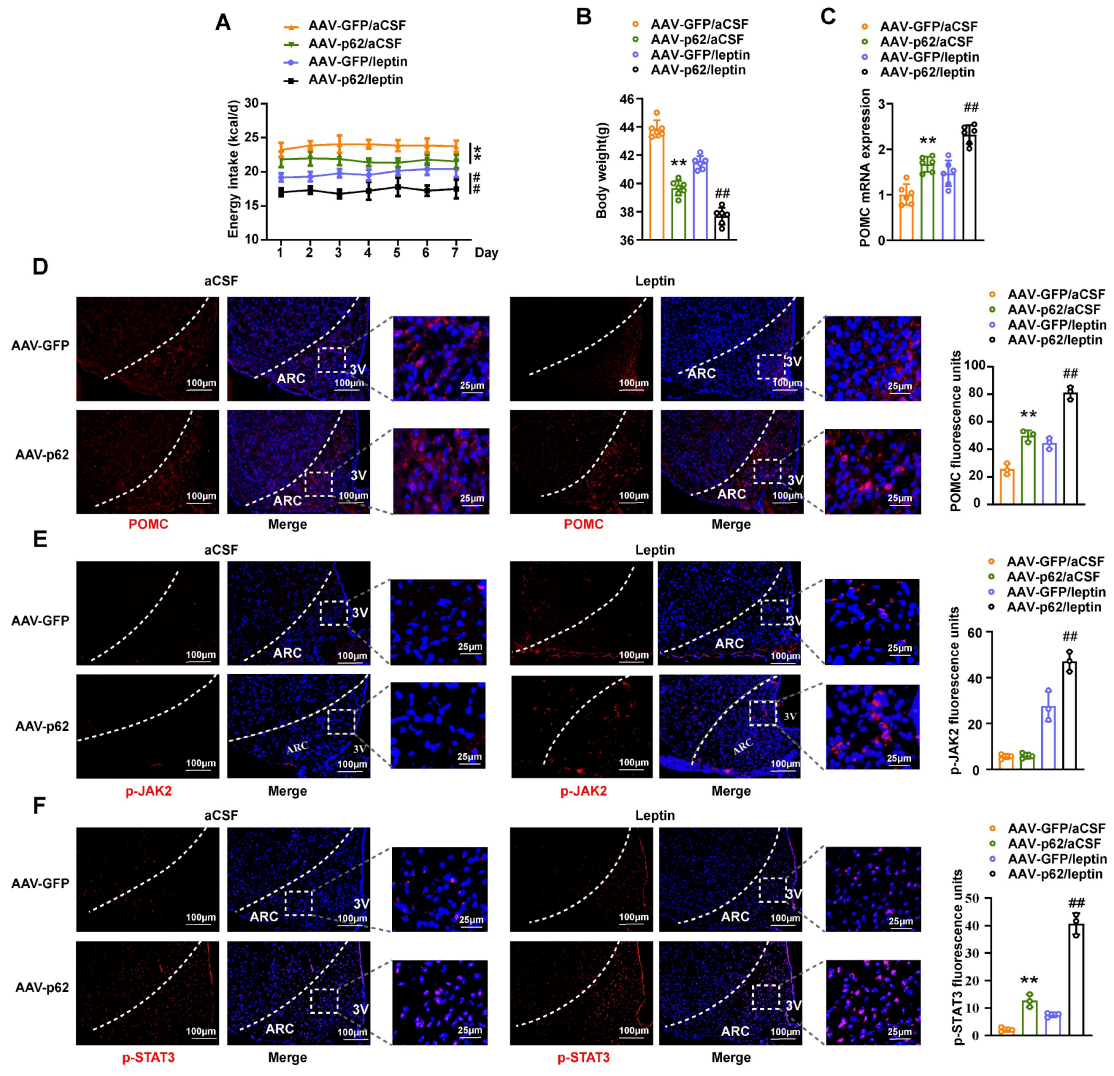
** Overexpression of p62 in the MBH alleviates obesity induced by leptin deficiency and promotes the anti-obesity effect of leptin in ob/ob mice.** Eight-week-old male ob/ob mice received bilateral MBH injection of AVV9-*p62/GFP*, followed by ICV leptin/vehicle infusion using an ALZET osmotic mini-pump for 7 days, as indicated in the Methods section. **(A)** Energy intake (n = 6-7 mice). **(B)** Body weight (n = 6-7 mice). **(C)** POMC mRNA expression in the hypothalamus (n = 6 mice). **(D)** IF staining of POMC (left) and RFU (right) in the ARC (n = 3 mice). **(E)** IF staining of phosphorylated JAK2 (left) and RFU (right) in the ARC (n = 3mice). **(F)** IF staining of STAT3 phosphorylation (left) and RFU (right) in the ARC (n = 3 mice). ARC, arcuate nucleus; 3V, third cerebral ventricle; aCSF, artificial cerebrospinal fluid; RFU, relative fluorescent units. Data are expressed as the mean ± SD. *p < 0.05, **p < 0.01 *vs.* GFP/aCSF; ^#^ *p* < 0.05, ^##^ *p* < 0.01 *vs*. GFP/leptin.

Expanding on our foundational findings, wherein p62 operates *via* the leptin/JAK2/STAT3 pathway and also engages with STAT3, we further explored the dependence of p62 on leptin signaling. To investigate this, we bilaterally injected AAV9-*p62/GFP* into the MBH of ob/ob mice, a model of global leptin deficiency (Fig. S8A). Four weeks post-injection, these mice underwent intracerebral (ICV) leptin/artificial cerebrospinal fluid (aCSF) administration for seven days. Hypothalamic p62 overexpression in ob/ob mice reduced energy intake, BW, and improved energy metabolism and glucose responsiveness, confirming that central p62 still plays a metabolic regulatory role under leptin deficiency conditions (Fig. 7A and B; Fig. S8B-D). Furthermore, p62 overexpression in the hypothalamus of ob/ob mice also enhanced the metabolic regulation of leptin. Notably, overexpression of p62 increased POMC expression and p-STAT3 levels in the ARC of ob/ob mice. In addition, overexpression of p62 also enhanced leptin-induced POMC expression, as well as an increase in p-JAK2 and p-STAT3 levels in the ARC of these animals (Fig. 7C-F). These data from ob/ob mice indicate that the regulation of metabolism by central p62 signaling is independent of leptin but can also enhance its effect.

### Deletion of STAT3 in POMC neurons blocks the effects of p62 on energy expenditure and systemic metabolism

**Figure 8:**
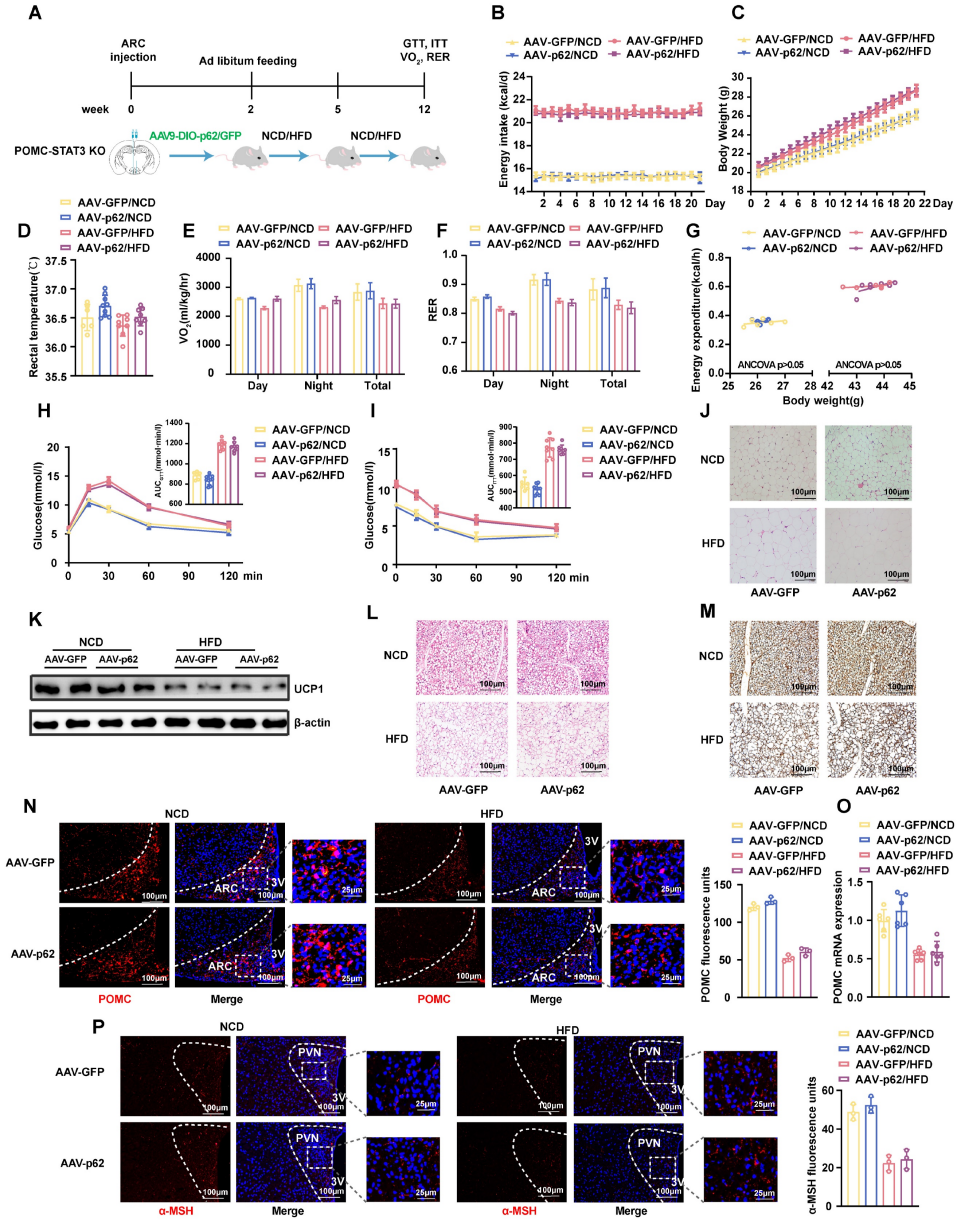
** Deletion of STAT3 in POMC neurons prevents the effects of p62 on energy expenditure and systemic metabolism. (A)** Schematic representation of the experimental procedure. Eight-week-old male POMC-STAT3 KO mice received bilateral ARC injection of AVV9-DIO-*p62/GFP* and were fed an NCD or HFD for 12 weeks as indicated in the Methods section. **(B)** Energy intake (n = 6-9 mice). **(C)** Body weight (n = 6-9 mice). **(D)** Rectal temperature (n = 6-9 mice). **(E)** 24-h oxygen consumption (Vo_2_) (n = 6-9 mice). **(F)** Respiratory exchange ratio (RER, V_CO2_/V_O2_) (n = 6-9 mice). **(G)** ANCOVA of the total energy expenditure versus body weight (n = 6-7 mice). **(H)** Blood glucose and AUC during the GTT (n = 6-9 mice). **(I)** Blood glucose and AUC during the ITT (n = 6-9 mice). **(J)** H&E staining of WAT (n = 3 mice). **(K)** UCP1 protein expression in WAT (n = 3 mice). **(L)** H&E staining of BAT (n = 3 mice).** (M)** UCP1 immunostaining in BAT (n = 3 mice). **(N)** IF staining of POMC (left) and RFU (right) in the ARC (n = 3 mice). (O) POMC mRNA expression in the hypothalamus (n = 6 mice). **(P)** IF staining for α-MSH and RFU (right) in the PVN (n = 3 mice). NCD, normal chow diet; HFD, high-fat diet; GTT, glucose tolerance test; ITT, insulin tolerance test; AUC, the area under the curve; ARC, arcuate nucleus; 3V, third cerebral ventricle; PVN, paraventricular nucleus; RFU, relative fluorescent units; WAT, white adipose tissue; BAT, brown adipose tissue. Data are expressed as the mean ± SD. *p < 0.05, **p < 0.01 *vs.* GFP/NCD; ^##^*p* < 0.01 *vs*. GFP/HFD.

To further elucidate the interrelationships among p62, STAT3, and FOXO1, we constructed different genetically engineered mouse models. Initially, we crossed STAT3^fl/fl^ mice with POMC-*Cre* mice to produce POMC-*STAT3* KO mice. We stereotactically injected AAV9-DIO-*p62/GFP* into the ARC of POMC-*STAT3* KO mice to produce POMC-*STAT3* KO/*p62* OE mice, and subjected the mice to either an NCD or HFD for 12 weeks (Fig. 8A). Our findings revealed that STAT3 KO diminished the effects of p62 overexpression on pivotal metabolic parameters, including energy intake, BW, energy expenditure, and glucose/lipid metabolism (Fig. 8B-M). Concurrently, IF staining confirmed that the effects of p62 overexpression on POMC and α-MSH expression is eliminated in the ARC and PVN of POMC-*STAT3* KO mice, respectively (Fig. 8N-P).

Similarly, we injected AAV9-DIO-*p62/GFP* into the ARC of POMC-*FOXO1* KO mice to explore whether p62 interacts with FOXO1 signaling (Fig. S9A). However, as expected, the deletion of FOXO1 did not alter the effects of p62 overexpression in the ARC on systemic metabolism and POMC expression (Fig. S9B-K). Collectively, these findings underscore that p62 primarily acts through the STAT3 pathway, not the FOXO1 pathway, to regulate metabolic and energy balance in the hypothalamus.

### The interaction between p62 and STAT3 regulates POMC expression

**Figure 9:**
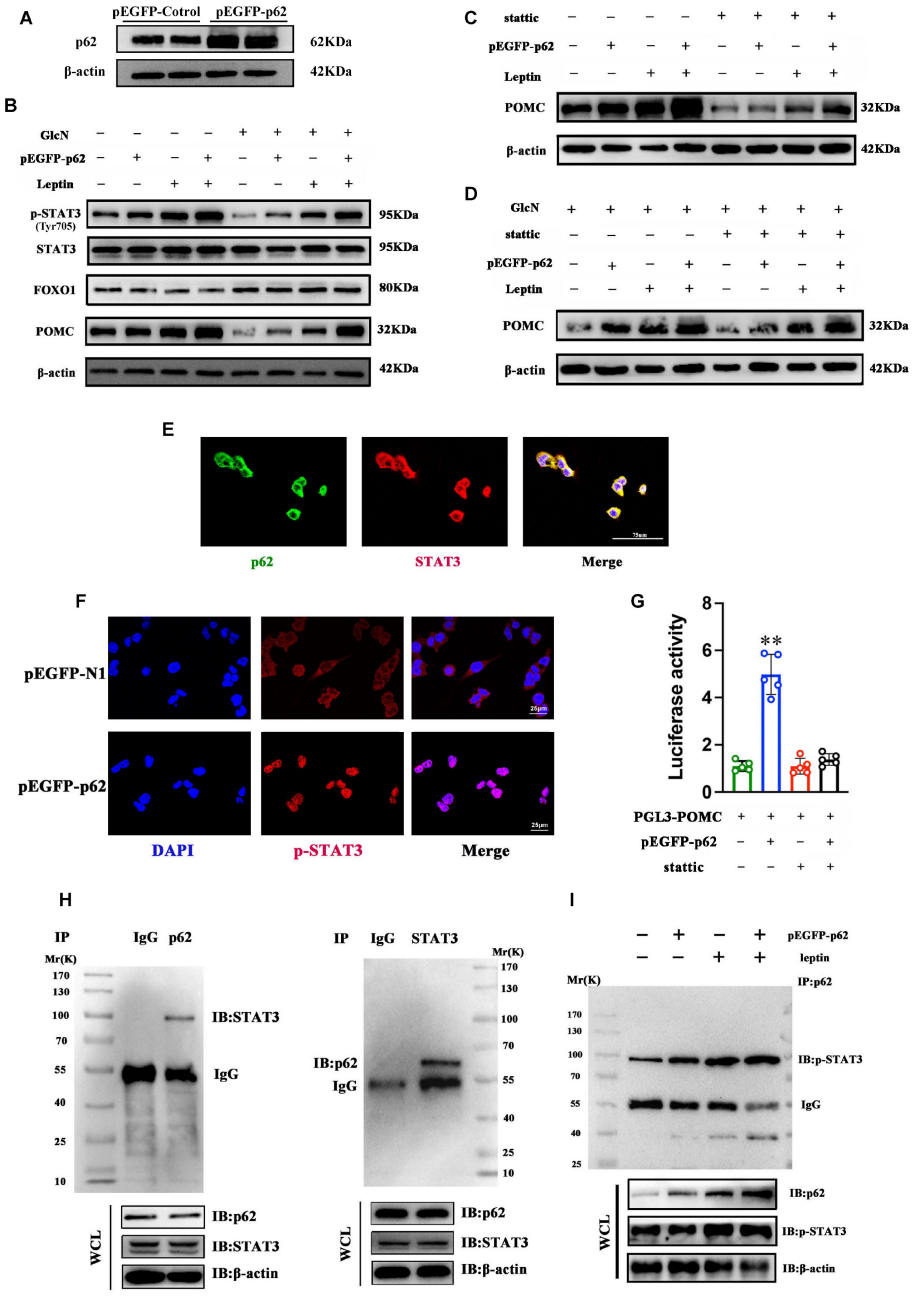
** Interaction between p62 and STAT3 regulates POMC expression and energy metabolism.** N2a cells were transfected with pEGFP-*p62* or pEGFP-*N1* for 48 h and incubated with GlcN for 18 h. The cells were then stimulated with or without leptin and/or Stattic for 30 min as indicated in the Methods section. **(A)** p62 protein expression. **(B)** Immunoblot analyses of total STAT3/pSTAT3 (Tyr705), FOXO1 and POMC protein expression in N2a cells treated with or without leptin. **(C)** Immunoblot analysis of POMC expression in N2a cells treated with or without leptin and/or Stattic. **(D)** Immunoblot analyses of POMC in N2a cells incubated with GlcN and treated with or without leptin or/and Stattic. **(E)** Representative confocal images showing the co-localization of p62 (green) and STAT3 (red) in N2a cells. **(F)** IF images showing STAT3 nuclear translocation. **(G)** N2a cells were transfected with PGL3-*POMC* or/and pEGFP-*p62* and then treated with or without Stattic. Promoter luciferase tests were performed. **(H)** Co-IP of STAT3 (left panel) and p62 (right panel) was performed with anti-p62 or anti-STAT3 antibody in N2a cells. **(I)** N2a cells were transfected with pEGFP-*p62*/*GFP* and then treated with or without leptin. Co-IP was performed with an anti-pSTAT3 antibody. GlcN, glucosamine; Stattic, a STAT3 inhibitor. Data are expressed as the mean ± SD (n = at least 3 independent experiments). **p < 0.01 *vs.* other treatments.

To further explore the cellular mechanisms underpinning the role of p62 in metabolism regulation, Neuro-2a (N2a) cells were transfected with a plasmid expressing p62 (pEGFP-*p62*) or a control vector. Transfection with pEGFP-*p62* significantly increased p62 and POMC expression and STAT3 phosphorylation but did not affect FOXO1 in N2a cells (Fig. 9A and B). We then examined the effect of STAT3 inhibition on p62-mediated POMC expression by using Stattic, a STAT3 inhibitor. In both insulin resistance (IR) and non-IR states, Stattic significantly inhibited p62-mediated POMC expression in N2a cells (Fig. 9C and D). Confocal microscopy revealed the co-localization of p62 and STAT3 in N2a cells (Fig. 9E). Notably, p62 expression also increased the translocation of STAT3 from the cytoplasm to the nucleus in these cells, further suggesting that p62 activates STAT3 signaling (Fig. 9F). Next, we used a POMC-luciferase reporter assay to examine the effects of p62 on POMC promoter activity. The results showed that p62 significantly enhanced POMC promoter activities, but stattic treatment abolished the effect of p62 (Fig. 9G), indicating that STAT3 is needed for p62-induced POMC transcription. Moreover, co-immunoprecipitation (co-IP) confirmed a molecular interaction between the p62 and STAT3 proteins (Fig. 9H). Finally, we sought to understand the impact of leptin on the interaction between p62 and STAT3 *in vitro.* A co-IP assay showed that leptin stimulation promoted p62 binding to STAT3 and that p62 increased leptin-mediated STAT3 phosphorylation in N2a cells (Fig. 9I). Collectively, these *in vitro* data support that p62 is an activator of STAT3 and facilitates leptin-mediated STAT3 phosphorylation.

## Discussion

Although many studies have shown that p62 is involved in energy homeostasis and glucose/lipid metabolism *in vivo,* only one study has reported that whole-brain p62 deficiency leads to hyperphagia and obesity 20; therefore, the role of p62 in whole-body metabolism and signal transduction pathways in specific neuronal populations has not been fully elucidated. In this study, we first performed snRNA-seq analysis on hypothalamic tissue from db/m and db/db mice and found that p62 was expressed in metabolism-related neurons, with significant differences in POMC neurons between obese and lean mice. We confirmed that p62 is highly expressed in hypothalamic metabolic-related nuclei and found that p62 signaling in POMC neurons regulates energy balance and glucose/lipid metabolism in both leptin-dependent and leptin-independent manners. Therefore, our data indicate that central p62 can independently and synergistically exert metabolic regulatory effects *via* leptin. Knockout or overexpression of p62 in POMC neurons promoted or inhibited weight gain, respectively, in both NCD- and HFD-fed mice, mainly by altering energy intake and energy expenditure. Importantly, even under normal diet conditions, specific p62 knockout in POMC neurons induced a significant obesity phenotype. This is very important because unless a HFD is fed, it is uncommon for a single gene knockout to lead to an obese phenotype. Mechanistically, p62 regulated JAK2/STAT3-POMC signaling by interacting with STAT3. This is the first study to report the function of p62 in metabolism-related neurons in terms of energy balance and glucose/lipid metabolism regulation, thus expanding our understanding of the role of p62 in obesity.

Similar to previous studies, this study also showed that p62 is expressed in the CNS 20. In particular, we found that the expression of p62 was high in the hypothalamic ARC and VMH, which is closely related to energy metabolism. The widespread expression of p62 in the metabolic-related nuclei of the hypothalamus provides clues for studying its role in central metabolic regulation. Hence, investigation into the roles played by hypothalamic p62 in the regulation of energy metabolism would be of great interest.

Although Harada *et al*. observed the effect of p62 on food intake and energy metabolism in whole-brain p62 KO mice, their work did not exclude the possible effect of p62 expression in peripheral tissue on the experimental results 20. It has been reported that hypothalamic signaling might be impacted by peripheral signaling 26, 27. Furthermore, this study cannot rule out the impact of global p62 expression deficiency on brain development. Therefore, to eliminate the interference of p62 expression in peripheral tissues, we re-established p62 expression in the MBH of the hypothalamus in p62-global-knockout (p62^-/-^) mice; that is, p62 was mainly expressed in the hypothalamus. Our results demonstrated that the selective expression of p62 in the MBH of p62^-/-^ mice fed either a NCD or HFD promoted energy expenditure and led to the acquisition of an anti-obesity phenotype. These findings confirmed that p62 expressed by the hypothalamus plays an important role in maintaining whole-body energy homeostasis and glucose/lipid metabolism, and these effects are independent of peripheral p62 expression. However, it is particularly important to accurately locate the neuronal population where p62 plays a major role.

Different neurons in the CNS have distinct functions. Therefore, p62 may play different roles in different neurons in the CNS, especially in the hypothalamus. Hypothalamic AgRP- and POMC-expressing neurons are considered the main leptin-responsive neurons 28. To investigate the role of p62 in metabolic-related neurons, we utilized gene editing technology to achieve specific knockout or overexpression of p62 in adult mouse POMC or AgRP neurons, thereby avoiding the impact of p62 deficiency on embryonic CNS development and other neural cells. Herein, we found that central p62 mainly acts on POMC neurons to regulate energy metabolism. Notably, independent of dietary conditions, adult-onset deletion of p62 in POMC-positive neurons led to the acquisition of an obesity phenotype, which was characterized by increased energy intake and BW, and reduced energy expenditure, while mice overexpressing p62 acquired a lean phenotype. Extending our investigation further, we observed that p62 also influences AgRP neurons, albeit predominantly under a HFD setting. Therefore, we believe that p62 expressed by POMC-positive neurons plays a primary role in maintaining energy metabolism* in vivo.*

The traditional view suggests that obesity is a result of a combination of environmental and genetic factors and has a polygenic effect. Several metabolic regulators, including protein tyrosine phosphatase-1B (PTP1B) 22, growth factor receptor-bound protein 10 (Grb10) 21, and thyroid transcription factor-1 (TTF-1) 29, have been identified. These regulators predominantly function in HFD environments where leptin resistance has developed. However, our findings challenge the traditional viewpoint that both environmental and genetic factors jointly contribute to obesity, emphasizing the important role of p62 in POMC neurons leading to obesity.

Hypothalamic ARC contains the most neurons that directly respond to leptin stimulation 30, and the leptin receptor (ObRb) is widely expressed in the hypothalamus 31. Leptin binds to ObRb expressed in hypothalamic neurons and regulates food intake and energy metabolism 28. Therefore, it is important to explore the functional association between p62 and leptin in the hypothalamus. Herein, we found that in leptin global knockout mice (ob/ob mice), hypothalamic p62 signaling partially reversed the obesity-phenotype caused by leptin deficiency, indicating that its anti-obesity effect is at least partially leptin-independent. Furthermore, p62 also enhanced the metabolic effect of leptin. However, it has been previously reported that leptin stimulation or the use of experimental supra-physiological ob/ob mice may affect the levels of other hormones 32. Therefore, we analyzed the metabolic phenotype of p62-specific KO in ObRb-expressing neurons and found that under both NCD and HFD conditions, p62 deficiency in these neurons exacerbated metabolic disorders and promoted the acquisition of an obesity phenotype. These results suggest that p62 may function, at least partially, as a downstream signaling molecule of leptin. Based on these findings, we speculate that hypothalamic p62, as a single gene, can regulate the metabolic phenotype in individuals on a normal diet, possibly through both leptin-dependent and leptin-independent mechanisms.

STAT3 plays an important role in mediating the metabolic effect of leptin 33. The phosphorylation of STAT3 is a known outcome of leptin receptor activation and a universally recognized indicator of leptin activity 34. STAT3 tyrosine phosphorylation has also been reported to increase after leptin injection in diet-induced obese mice 35. In this study, we found that the deletion of p62 in hypothalamic POMC-positive neurons attenuated STAT3 phosphorylation, STAT3 translocation from the cytoplasm to the nucleus and decreased POMC neuron excitability, while the overexpression of p62 in POMC neurons had the opposite effect. Importantly, in POMC-STAT3 KO mice, the effect of p62 was negligible, indicating that STAT3 mediates the regulation of energy homeostasis by p62.

Notably, at the molecular level, we found that p62 can interact with STAT3, and in the presence of leptin, the interaction between these two molecules is further enhanced. Therefore, we believe that p62 interacts with STAT3 at a specific location and enhances leptin signaling to regulate energy homeostasis. By further elucidating the molecular mechanism involved, we found that p62 can modulate POMC expression by interacting with STAT3 and thereby regulating peripheral metabolism.

Interestingly, recent findings suggest that leptin-mediated STAT3 activation is dispensable for the leptin response in AgRP neurons 36, 37. Consistent with these findings, in STAT3-deficient mice, AgRP neurons do not exhibit any significant alterations in AgRP mRNA levels 38. These reports provide a potential explanation for the lack of metabolic phenotype changes observed in mice with p62 overexpression in AgRP neurons under NCD conditions. However, this phenomenon still requires further investigation.

In summary, our study highlights the pivotal role of hypothalamic p62 in adult-onset obesity, which is notably independent of environmental influences such as a HFD. In addition, we revealed the potential of p62 signaling in POMC neurons to regulate metabolism and emphasized its dual role in promoting POMC transcription through interaction with STAT3 and enhancing leptin sensitivity. These data will undoubtedly help to understand the pathological mechanism underlying obesity and indicate that hypothalamic p62 may be a central target for the treatment of obesity and metabolic diseases.

## Methods

### Experimental animals and genotyping

Male C57BL/6J (WT) mice aged 8 weeks were purchased from Jicuiyaokang Biotechnology Co., Ltd. (Jiangsu, China). POMC*-Cre* mice, tamoxifen-inducible POMC*-Cre* mice (POMC-*Cre*^ER^), and AI9 (tdTomato) reporter mice were generously provided by Dr. Guo (Institute for Nutritional Sciences, Shanghai Institutes for Biological Sciences, Chinese Academy of Sciences, Shanghai, China). P62 global knockout (P62^-/-^) mice, p62^flox/flox^ mice, and ob/ob mice were generated or purchased from the Model Animal Center of Nanjing University (Nanjing, China). FOXO1^fl/fl^ mice were kindly provided by Dr. Li and Dr. Deng (Third Military Medical University, Chongqing, China). Stat3^fl/fl^ mice were provided by Dr. Liu (Department of Pathogen Biology, School of Basic Medicine, Huazhong University of Science and Technology, Wuhan, China). Leptin receptor neuron (ObRb)-Cre mice were purchased from Cyagen Biotechnology Co., Ltd (Jiangsu, China). All mice were bred on the C57BL/6J background and housed under a 12-h light/dark cycle at 25°C, with water and food available ad libitum.

To visualize POMC- or ObRb-expressing neurons under a fluorescence microscope, AI9 (tdTomato) reporter mice were crossed with POMC-*Cre*^ER^ or ObRb-*Cre* mice to generate mice with POMC or ObRb neurons labeled with tdTomato. P62^fl/fl^ mice were crossed with ObRb-*Cre*:tdTomato mice or POMC-*Cre*^ER^:tdTomato mice to generate ObRb-p62 KO mice and POMC-p62 KO mice, respectively, with tdTomato. The effectiveness of p62 knockout on POMC or ObRb cells was confirmed by evaluating the expression of tdTomato with a fluorescence microscopy.

For the signaling pathway study, FOXO1^fl/fl^ mice or Stat3^fl/fl^ mice were crossed with POMC-*Cre*^ER^ mice to generate POMC-FOXO1 KO mice or POMC-STAT3 KO mice, respectively. All genetically engineered mice were generated and identified as previously reported 39.

To establish tamoxifen-induced gene knockout mice, 8-week-old male POMC-p62 KO, POMC-FOXO1 KO, or POMC-Stat3 KO mice were intraperitoneally injected with tamoxifen (0.15 g/kg; Sigma-Aldrich, St. Louis, MO) for 6 days to generate mice with adult-onset gene deletion in POMC neurons.

For diet-induced obesity, mice were fed a HFD with 60% kcal from fat (D12492, Research Diets, New Brunswick, NJ) or a NCD for 12 weeks beginning at eight weeks of age. Pair-feeding tests were performed by feeding control animals the same amounts of food as experimental animals. All procedures were approved by the Animal Experimentation Ethics Committee, Chongqing Medical University.

### Single-nucleus RNA Sequencing (sn-RNA Seq)

Ten-week-old male db/db and db/m mice were divided into two groups (n = 3 for each group). Mice were euthanized, and their brains were quickly extracted for dissection to obtain hypothalamic tissue. To prepare a single cell suspension, hypothalamic tissues in ice-cold lysis buffer [0.25 M sucrose, 5 × 10 ^-3^ M CaCl_2_, 3 × 10 ^-3^ M MgAC_2_, 10 × 10 ^- 3^ M Tris HCl (pH 8.0), 0.1 × 10^-3^ M EDTA, 1x protease inhibitor, and 1 U μL^-1^ RiboLock RNase inhibitor] were homogenized. Next, the homogenates were filtered through a 70 × 10^-6^ M cell strainer to collect the nuclear fraction. The nuclear fraction was mixed with 50% iodixanol and placed on top of a 30% iodixanol solution. Then, the solution was centrifuged for 20 min at 10 000×g. The nuclei were collected from 30% iodixanol, resuspended in nuclear washing buffer and resuspension buffer, and then were centrifuged at 4℃ for 5 min. The nuclei were filtered by a cell filter to remove cell fragments. Finally, the nuclear concentration was adjusted to 700~1200 nuclei μL^-1^. The nuclei were checked using the 10X Chromium platform. Reverse transcription, cDNA amplification, and library preparation were performed according to the manufacturer's protocol.

For data preprocessing, raw data were analyzed using Cell Ranger (version 3.1.0) and compared with the reference genome. We used the Seurat R package (version 4.3.0) to process the information. The criteria for cell screening were as follows: l) the number of genes identified in a single cell ranged from 200 to 5500, 2) the total number of cells with Unique Molecular Identifier (UMl) 1000-16000 was retained; and 3) the proportion of mitochondrial gene expression in single cells was less than 5%. These data were subsequently visualized* via t*-distributed stochastic neighbor embedding (tSNE) and cells with the same expression pattern were clustered. We annotated the cell cluster by using the marker gene and screened neuronal cells and then further analyzed the localization and expression of p62 (SQSTM1) in neurons.

### Stereotactic surgery and microinjection of adeno-associated virus (AAV)

Mice were anesthetized with isoflurane and stereotaxic surgery was performed, as previously described 40. WT, p62^-/-^ and ob/ob mice were bilaterally injected with AAV expressing p62 (AAV9-*p62*) or AAV9-*GFP* (2×10^12^ pfu/ml) into the MBH (-1.6 mm from bregma; ± 0.3 mm from midline; -5.80 mm from the dorsal surface) at a volume of 200 nL/side. To specifically establish p62 expression in POMC neurons, POMC-Cre^ER^, POMC-Stat3 KO and POMC-FOXO1 KO mice were bilaterally injected with Cre-dependent AAV expressing p62/GFP (AAV9-DIO-*p62/GFP*, 2×10^12^ pfu/ml) into the ARC (-1.4 mm from bregma; ± 0.3 mm from midline; -5.90 mm from dorsal surface) 41. After recovery from the stereotactic surgery, all mice were fed a NCD or HFD. Energy intake and BW were recorded daily or weekly. The localization and effectiveness of MBH or ARC injection were confirmed by the expression of GFP with a fluorescence microscope. For the leptin signaling study, mice were intraperitoneally (i.p.) injected with leptin (1.5 mg /kg per injection, PeproTech, Rehovot, Israel) or saline (0.9% NaCl) for 3 days.

### Intracerebroventricular (ICV) cannulation and treatment

ICV cannulation and osmotic pump implantation were performed as previously reported 30, 42. Briefly, ob/ob mice were anesthetized and a cannula was implanted into the ICV (AP: -0.50 mm, ML: ±1.3 mm, DV: -2.3 mm). For the ICV infusion of leptin, an osmotic pump (Model 1007D, Alzet) was implanted subcutaneously through a catheter connected to the cannula. Leptin (454 ng/μl) or aCSF was continuously infused* via* the osmotic pump for 7 days after post-surgery recovery. Metabolic indices such as energy intake and BW were measured.

### Metabolic analyses

The mice were acclimated in the metabolic chamber for 2 days before the experiment. BW, food intake, and rectal temperature were measured at the indicated time points and durations. V_O2_ and RER (V_CO2_/V_O2_) were measured by indirect calorimetry 40.

### GTT and ITT

For the GTT, after overnight fasting, the mice were intraperitoneally injected with 20% glucose. Blood glucose was measured before and 15, 30, 60, and 120 min after the injection. For the ITT, the mice were fasted for 4 h, and blood glucose was measured at 0, 15, 30, 60, and 120 min after intraperitoneal injection of insulin (0.5 U/kg) 40, 43.

### Histopathology and IHC staining

The tissues were fixed in 4% paraformaldehyde overnight, embedded in paraffin, cut into 5 μM histological sections, and stained with H&E 1. Frozen tissue sections were stained with Oil Red O as previously described 1. For IHC staining, after the tissue sections were dewaxed and rehydrated, the antigen was recovered for 10 min in citric acid buffer at 90°C. The tissue sections were blocked with 5% goat serum in 0.1 M PBS and incubated overnight with anti-p62 (1:500, ab109012, Abcam, Cambridge, UK) and anti-UCP1 antibodies (1:500, ab10983, Abcam, Cambridge, UK) at 4°C. The sections were then washed with PBS and incubated with an HRP-conjugated secondary antibody (1: 1000, SAP-9100, ZSGB-BIO, China) for visualization 41.

### IF staining

IF staining was performed as previously reported with minor modifications 44, 45. Briefly, mice were perfused with 4% paraformaldehyde. Brains were dissected and cut into 7 μM sections. Brain sections and cultured N2a cells were incubated with primary antibody at 4ºC overnight and secondary antibodies at room temperature for 120 min. Counterstaining and mounting were performed with mounting medium containing DAPI. For IF staining of STAT3 phosphorylation, the sections were eluted with 1× PBS at each step of successive blocking with 1.0% H_2_O_2_/1.0% NaOH in H_2_O (20 min), 0.3% glycine (10 min), 0.03% SDS (10 min) and 0.2% 5% normal goat serum/0.5% Triton X-100 in PBS (30 min) at room temperature. The sections were then incubated with an anti-p-STAT3 antibody for 48 h at 4℃ 46. The primary antibodies used were anti-c-Fos (1: 300, ab222699, Abcam), anti-p-STAT3(Y705) (1: 300, #9145, CST), anti-STAT3 (1:300, #9139, CST), anti-POMC (1: 300, #23499, CST), anti-AgRP (1: 300, ab254558, Abcam), anti-FOXO1 (1: 500, #2880, Abcam), anti-p62 (1: 300, ab109012, Abcam) anti-p-JAK2 (1:100, ab32101, Abcam) and anti-α-MSH (1:100, ab120189, Abcam). The secondary antibodies used were goat anti-mouse IgG (1: 300, A-21426 and A-21121, Invitrogen), goat anti-rabbit IgG (1:300, A32732 and A-11034, Invitrogen), and donkey anti-rabbit IgG (1: 300, A32790, Invitrogen).

### Cell culture and treatments

N2a cells were maintained in DMEM with 10% FBS and 1% antibiotics as described 47. After serum starvation, N2a cells were infected with pEGFP-*p62* or pEGFP-N1 for 48 h. To induce IR, N2a cells were incubated with glucosamine (GlcN, 18 mM) or 1% BSA as a control in serum-free medium for 20 h. Then, the cells were treated with or without leptin (30 nM) or Stattic (a STAT3 inhibitor, 15 μM) for 30 min. Cell lysates were collected and stored at -80°C for further analysis.

### Luciferase assays

To investigate the effect of p62 activity on the POMC promoter, N2a cells were seeded and cultured in a 24-well plate for 12 h. When the cells reached 60-70% confluence, the POMC-luciferase reporter plasmid (pGL3-POMC, -2000 to 0 bp) and/or pEGFP-p62 and/or empty plasmids (pEGFP-N1) were co-transfected into cells for 24 h. The cells were then treated with or without stattic for 30 min. Luciferase activity was measured using the Dual-Luciferase® Reporter Assay System as previously reported 1.

### Quantitative RT‒PCR (qRT‒PCR)

qRT-PCR was performed as previously described 48. Briefly, TRIzol reagent (Takara Biomedical Technology Co., Ltd, Beijing) was used to extract total RNA from hypothalamic tissues. Reverse transcriptase was used to synthesize complementary DNA. After the cDNA, primer and master mix were combined, qRT‒PCR was performed with the CFX ConnectTM Optics Module (Bio-Rad, California, USA). The primer pairs used were Pomc R: CATTAGGCTTGGAGCAGGTC; F: TCTTGATGATGGCGTTCTTG and β-actin R: GGCATAAACGCAGAGCATTCCTG; F: CAGTGTCCATCCTCTGAGTAGC.

### Protein expression

Western blotting was performed using appropriate primary and secondary antibodies, including anti-p62 (1:1000, ab109012, Abcam), anti-UCP1 (1: 600, ab10983, Abcam), anti-POMC (1:800, #23499, CST), anti-FOXO1 (1:1000, #2880, Abcam), anti-STAT3 (1:1000, #9139, CST)/p-STAT3 (Y705) (1: 1000, #9145, CST) and anti-β-actin (Sigma- Aldrich) antibodies as previously described 49.

### Co-immunoprecipitation (Co-IP) assay

For the Co-IP test, N2a cells were harvested and washed twice with ice-cold PBS. Then, the cells were digested with 300 μl of IP lysate for 5 min. Cells were collected and centrifuged at 12000 rpm and 4°C for 10 min, and the supernatant was collected. Then, 500 μL of supernatant was placed in Protein G-Agarose beads and gently shaken at 4°C for 1 h. The supernatant was divided into two parts, and 2 μL of antibodies (anti-p62 or anti-Stat3) or IgG (control) was added at 4℃ overnight with constant rotation. The proteins were separated *via* western blotting as previously described 39.

### Statistical analyses

Statistical analyses were conducted using either SPSS software (version 25.0) or Prism software (version 8.0). All data are expressed as the mean ± SD. The differences were evaluated by one or two-way ANOVA followed by Bonferroni's post hoc tests analysis or a two-tailed unpaired Student's t-test, as appropriate. *P* values less than 0.05 were considered statistically significant.

## Supplementary Material

Supplementary figures.

## Abbreviations

POMC: pro-opiomelanocortin
NCD: normal chow
HFD: high-fat diet
CNS: central nervous system
ARC: the arcuate nucleus
VMH: ventromedial hypothalamus
AgRP: agouti-related protein
NPY: neuropeptide Y
NAFLD: nonalcoholic fatty liver disease
T2D: type 2 diabetes
IHC: immunohistochemical
MBH: mediobasal hypothalamus
IF: immunofluorescence
RFU: relative fluorescent units
BW: body weight
GTT: glucose tolerance test
ITT: insulin tolerance test
BAT: brown adipose tissue
α-MSH: α-melanocyte-stimulating hormone
p-STAT3: phosphorylated STAT3
FOXO1: forkhead box protein O1
ObRb: leptin receptor neuron
aCSF: artificial cerebrospinal fluid
N2a cells: Neuro-2a
co-IP: coimmunoprecipitation
P62^-/-^: p62 global knockout
V_O2_: 24-h oxygen consumption
V_CO2_: carbon dioxide produced
RER: respiratory exchange ratio

## References

[1] Zhang C, Luo X, Chen J, Zhou B, Yang M, Liu R (2019). Osteoprotegerin Promotes Liver Steatosis by Targeting the ERK-PPAR-γ-CD36 Pathway. Diabetes.

[2] Nagao M, Esguerra JLS, Asai A, Ofori JK, Edlund A, Wendt A (2020). Potential Protection Against Type 2 Diabetes in Obesity Through Lower CD36 Expression and Improved Exocytosis in β-Cells. Diabetes.

[3] Spiegelman BM, Flier JS (2001). Obesity and the regulation of energy balance. Cell.

[4] Morton GJ, Cummings DE, Baskin DG, Barsh GS, Schwartz MW (2006). Central nervous system control of food intake and body weight. Nature.

[5] Cowley MA, Smart JL, Rubinstein M, Cerdán MG, Diano S, Horvath TL (2001). Leptin activates anorexigenic POMC neurons through a neural network in the arcuate nucleus. Nature.

[6] Myers MG Jr, Olson DP (2012). Central nervous system control of metabolism. Nature.

[7] Sisley S, Sandoval D (2011). Hypothalamic control of energy and glucose metabolism. Rev Endocr Metab Disord.

[8] Cansell C, Denis RG, Joly-Amado A, Castel J, Luquet S (2012). Arcuate AgRP neurons and the regulation of energy balance. Front Endocrinol (Lausanne).

[9] Belgardt BF, Brüning JC (2010). CNS leptin and insulin action in the control of energy homeostasis. Ann N Y Acad Sci.

[10] Yang L, McKnight GS (2015). Hypothalamic PKA regulates leptin sensitivity and adiposity. Nat Commun.

[11] Moscat J, Diaz-Meco MT, Wooten MW (2007). Signal integration and diversification through the p62 scaffold protein. Trends Biochem Sci.

[12] Puls A, Schmidt S, Grawe F, Stabel S (1997). Interaction of protein kinase C zeta with ZIP, a novel protein kinase C-binding protein. Proc Natl Acad Sci U S A.

[13] Joung I, Strominger JL, Shin J (1996). Molecular cloning of a phosphotyrosine-independent ligand of the p56lck SH2 domain. Proc Natl Acad Sci U S A.

[14] Shin J (1998). P62 and the sequestosome, a novel mechanism for protein metabolism. Arch Pharm Res.

[15] Moscat J, Diaz-Meco MT (2009). p62 at the crossroads of autophagy, apoptosis, and cancer. Cell.

[16] Komatsu M, Kageyama S, Ichimura Y (2012). p62/SQSTM1/A170: physiology and pathology. Pharmacol Res.

[17] Lee SJ, Pfluger PT, Kim JY, Nogueiras R, Duran A, Pagès G (2010). A functional role for the p62-ERK1 axis in the control of energy homeostasis and adipogenesis. EMBO Rep.

[18] Geetha T, Zheng C, Vishwaprakash N, Broderick TL, Babu JR (2012). Sequestosome 1/p62, a scaffolding protein, is a newly identified partner of IRS-1 protein. J Biol Chem.

[19] Müller TD, Lee SJ, Jastroch M, Kabra D, Stemmer K, Aichler M (2013). p62 links β-adrenergic input to mitochondrial function and thermogenesis. J Clin Invest.

[20] Harada H, Warabi E, Matsuki T, Yanagawa T, Okada K, Uwayama J (2013). Deficiency of p62/Sequestosome 1 causes hyperphagia due to leptin resistance in the brain. J Neurosci.

[21] Liu H, He Y, Bai J, Zhang C, Zhang F, Yang Y (2023). Hypothalamic Grb10 enhances leptin signalling and promotes weight loss. Nat Metab.

[22] Dodd GT, Decherf S, Loh K, Simonds SE, Wiede F, Balland E (2015). Leptin and insulin act on POMC neurons to promote the browning of white fat. Cell.

[23] Vohra MS, Benchoula K, Serpell CJ, Hwa WE (2022). AgRP/NPY and POMC neurons in the arcuate nucleus and their potential role in treatment of obesity. Eur J Pharmacol.

[24] Chen Y, Lin YC, Kuo TW, Knight ZA (2015). Sensory detection of food rapidly modulates arcuate feeding circuits. Cell.

[25] Padilla SL, Carmody JS, Zeltser LM (2010). Pomc-expressing progenitors give rise to antagonistic neuronal populations in hypothalamic feeding circuits. Nat Med.

[26] Schneeberger M, Dietrich MO, Sebastián D, Imbernón M, Castaño C, Garcia A (2013). Mitofusin 2 in POMC neurons connects ER stress with leptin resistance and energy imbalance. Cell.

[27] Kleinridders A, Schenten D, Könner AC, Belgardt BF, Mauer J, Okamura T (2009). MyD88 signaling in the CNS is required for development of fatty acid-induced leptin resistance and diet-induced obesity. Cell Metab.

[28] Deng J, Yuan F, Guo Y, Xiao Y, Niu Y, Deng Y (2017). Deletion of ATF4 in AgRP Neurons Promotes Fat Loss Mainly via Increasing Energy Expenditure. Diabetes.

[29] Park BS, Kang D, Kim KK, Jeong B, Lee TH, Park JW (2022). Hypothalamic TTF-1 orchestrates the sensitivity of leptin. Mol Metab.

[30] Xu J, Bartolome CL, Low CS, Yi X, Chien CH, Wang P (2018). Genetic identification of leptin neural circuits in energy and glucose homeostases. Nature.

[31] Scott MM, Lachey JL, Sternson SM, Lee CE, Elias CF, Friedman JM (2009). Leptin targets in the mouse brain. J Comp Neurol.

[32] Sun JS, Yang DJ, Kinyua AW, Yoon SG, Seong JK, Kim J (2021). Ventromedial hypothalamic primary cilia control energy and skeletal homeostasis. J Clin Invest.

[33] Vaisse C, Halaas JL, Horvath CM, Darnell JE Jr, Stoffel M, Friedman JM (1996). Leptin activation of Stat3 in the hypothalamus of wild-type and ob/ob mice but not db/db mice. Nat Genet.

[34] Pan WW, Myers MG Jr (2018). Leptin and the maintenance of elevated body weight. Nat Rev Neurosci.

[35] Münzberg H, Flier JS, Bjørbaek C (2004). Region-specific leptin resistance within the hypothalamus of diet-induced obese mice. Endocrinology.

[36] Morrison CD, Morton GJ, Niswender KD, Gelling RW, Schwartz MW (2005). Leptin inhibits hypothalamic Npy and Agrp gene expression via a mechanism that requires phosphatidylinositol 3-OH-kinase signaling. Am J Physiol Endocrinol Metab.

[37] Münzberg H, Jobst EE, Bates SH, Jones J, Villanueva E, Leshan R (2007). Appropriate inhibition of orexigenic hypothalamic arcuate nucleus neurons independently of leptin receptor/STAT3 signaling. J Neurosci.

[38] Kaelin CB, Gong L, Xu AW, Yao F, Hockman K, Morton GJ (2006). Signal transducer and activator of transcription (stat) binding sites but not stat3 are required for fasting-induced transcription of agouti-related protein messenger ribonucleic acid. Mol Endocrinol.

[39] Lai Y, Zhao A, Tan M, Yang M, Lin Y, Li S (2020). DOCK5 regulates energy balance and hepatic insulin sensitivity by targeting mTORC1 signaling. EMBO Rep.

[40] Zhou M, Xu X, Wang H, Yang G, Yang M, Zhao X (2020). Effect of central JAZF1 on glucose production is regulated by the PI3K-Akt-AMPK pathway. Faseb j.

[41] Yuan F, Jiang H, Yin H, Jiang X, Jiao F, Chen S (2020). Activation of GCN2/ATF4 signals in amygdalar PKC-δ neurons promotes WAT browning under leucine deprivation. Nat Commun.

[42] Qu H, Miao T, Wang Y, Tan L, Huang B, Zhang L (2021). Dedicator of Cytokinesis 5 Regulates Keratinocyte Function and Promotes Diabetic Wound Healing. Diabetes.

[43] Yang M, Wang J, Wu S, Yuan L, Zhao X, Liu C (2017). Duodenal GLP-1 signaling regulates hepatic glucose production through a PKC-δ-dependent neurocircuitry. Cell Death Dis.

[44] Deng Y, Xiao Y, Yuan F, Liu Y, Jiang X, Deng J (2018). SGK1/FOXO3 Signaling in Hypothalamic POMC Neurons Mediates Glucocorticoid-Increased Adiposity. Diabetes.

[45] Kong D, Tong Q, Ye C, Koda S, Fuller PM, Krashes MJ (2012). GABAergic RIP-Cre neurons in the arcuate nucleus selectively regulate energy expenditure. Cell.

[46] Tsunekawa T, Banno R, Mizoguchi A, Sugiyama M, Tominaga T, Onoue T (2017). Deficiency of PTP1B Attenuates Hypothalamic Inflammation via Activation of the JAK2-STAT3 Pathway in Microglia. EBioMedicine.

[47] Kaushik S, Rodriguez-Navarro JA, Arias E, Kiffin R, Sahu S, Schwartz GJ (2011). Autophagy in hypothalamic AgRP neurons regulates food intake and energy balance. Cell Metab.

[48] Qiu S, Wu Q, Wang H, Liu D, Chen C, Zhu Z (2024). AZGP1 in POMC neurons modulates energy homeostasis and metabolism through leptin-mediated STAT3 phosphorylation. Nat Commun.

[49] Li Y, Tian M, Yang M, Yang G, Chen J, Wang H (2020). Central Sfrp5 regulates hepatic glucose flux and VLDL-triglyceride secretion. Metabolism.

