# Supplementary material for: Hypothalamic P62 (SQSTM1) regulates energy balance by modulating leptin signaling
Source: Theranostics. 2024 Oct 7;14(17):6605–24. doi: 10.7150/thno.96480 (PMC11519807; doi:10.7150/thno.96480)
Supplement: Supplementary file 1 — Supplementary figures. [file thnov14p6605s1.pdf]

Supplementary Figures

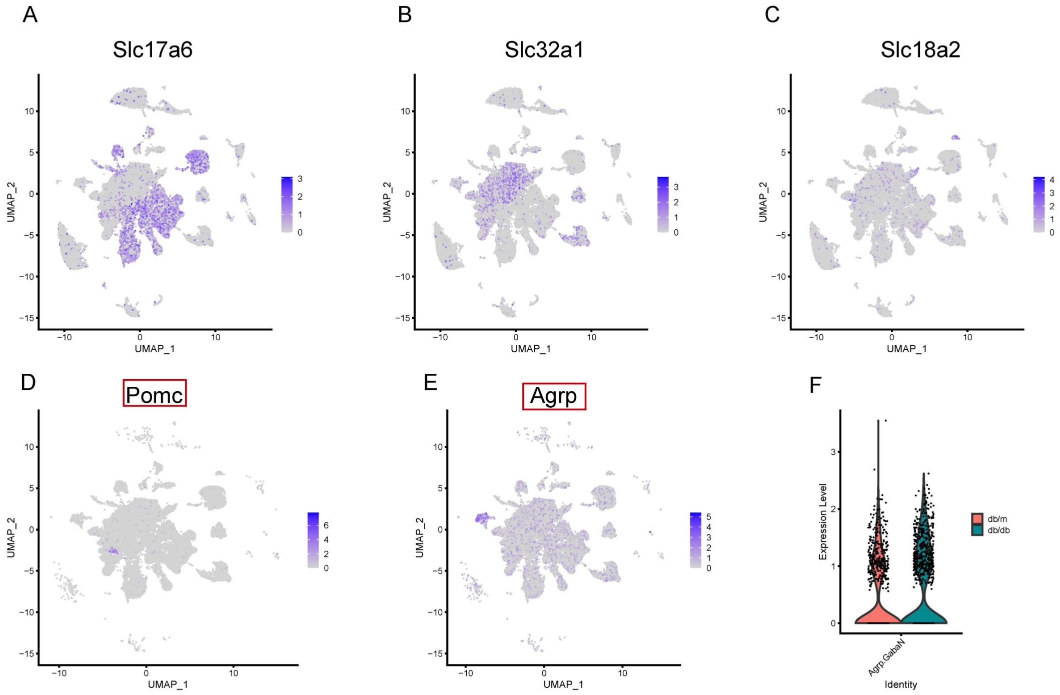

Figure S1 Molecular markers of neuronal clustering. (A) UMAP expression of the neuronal marker Slc17a6 in the hypothalamus (n = 3 mice). **(B)** UMAP expression of neuronal marker Slc32a1 clusters in the hypothalamus (n = 3 mice). **(C)** UMAP expression of neuronal marker Slc18a2 clusters in the hypothalamus (n = 3 mice). **(D)** UMAP expression of Pomc clusters in the hypothalamus (n = 3 mice). **(E)** UMAP expression of Agrp clusters in the hypothalamus (n = 3 mice). **(F)** Expression of P62 in db/db and db/m groups of AgRP neurons (n = 3 mice).

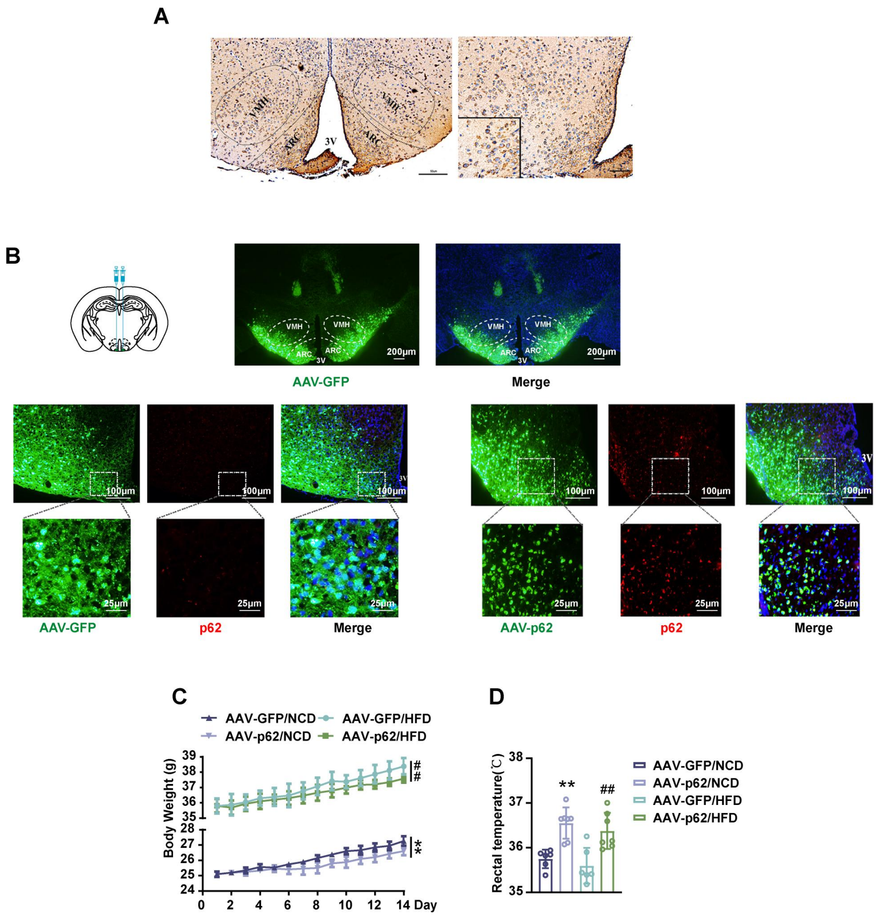

Figure S2 Body weight and rectal temperature in pair-fed mice. Eight-week-old male WT mice were fed an NCD or HFD for 12 weeks and received MBH injection of AVV9-*p62/GFP* as indicated in the Methods. **(A)** P62 immunostaining in the ARC and VMH of C57BL/6J (WT) mice. **(B)** Distribution of the fluorescent reporter GFP (green) in the hypothalamus (upper panel) and immunofluorescence (IF) staining of p62 (red) and GFP (green) in MBHs (bottom panels, left for AAV-*GFP*; right for AAV-*p62*) (n = 3 mice). **(C)** Daily body weight (n = 6-7 mice). **(D)** Rectal temperature (n = 6-7 mice). ARC, arcuate nucleus; 3V, third cerebral ventricle; VMH, ventromedial hypothalamus; NCD, normal chow diet; HFD, high-fat diet. Data are expressed as the mean ± SD. $^{**}p < 0.01$ *vs.* GFP/NCD; $^{\#\#}p < 0.01$ *vs.* GFP/HFD.

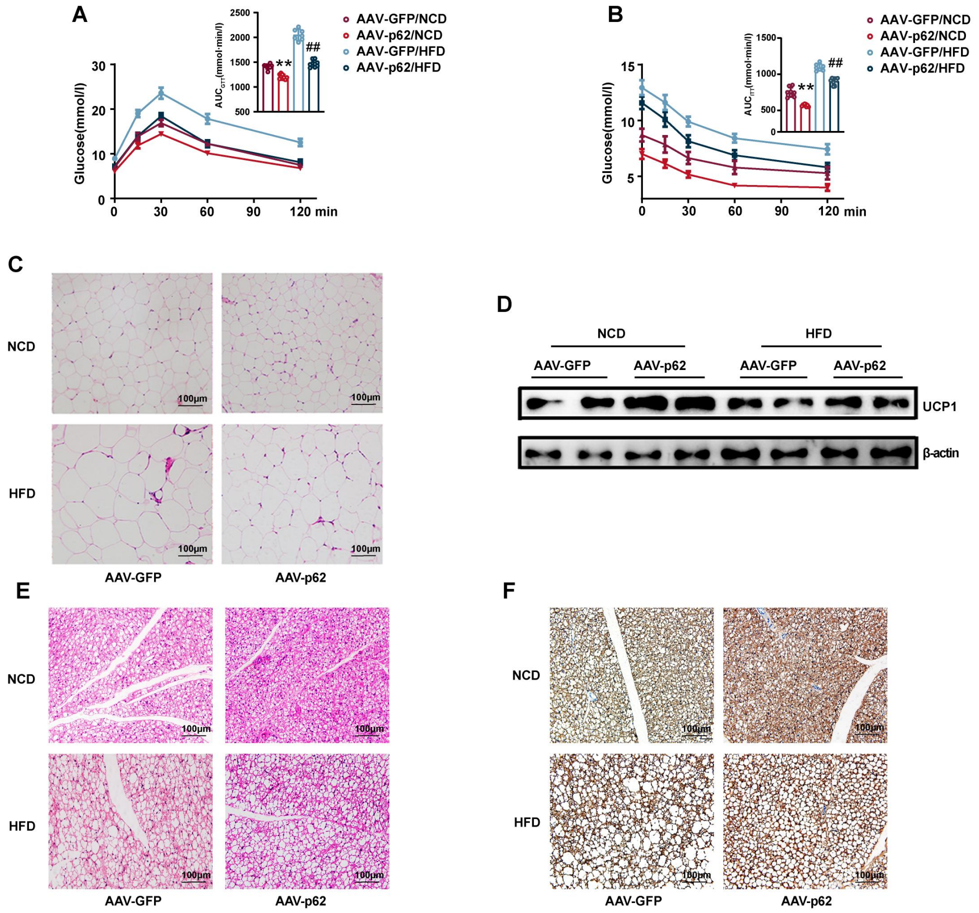

Figure S3 The re-expression of p62 in the hypothalamus improved glucose and lipid metabolism in p62$^{-/-}$ mice. Eight-week-old male p62$^{-/-}$ mice were fed an NCD or HFD for 18 weeks and received bilateral MBH injection of AVV9-*p62/GFP* at week 12 as indicated in the Methods. **(A and B)** Blood glucose and AUC during the GTT (A) and ITT (B) (n = 7-9 mice). **(C)** H&E staining in WAT (n = 3 mice). **(D)** UCP1 protein expression in WAT (n = 3 mice). **(E)** H&E staining in BAT (n = 3 mice). **(F)** UCP1 immunostaining in BAT (n = 3 mice). NCD, normal chow diet; HFD, high-fat diet; GTT, glucose tolerance test; ITT, insulin tolerance test; AUC, the area under the curve; WAT, white adipose tissue; BAT, brown adipose tissue. Data are expressed as the mean ± SD. $^{**}p < 0.01$ *vs.* GFP/NCD; $^{\#\#}p < 0.01$ *vs.* GFP/HFD.

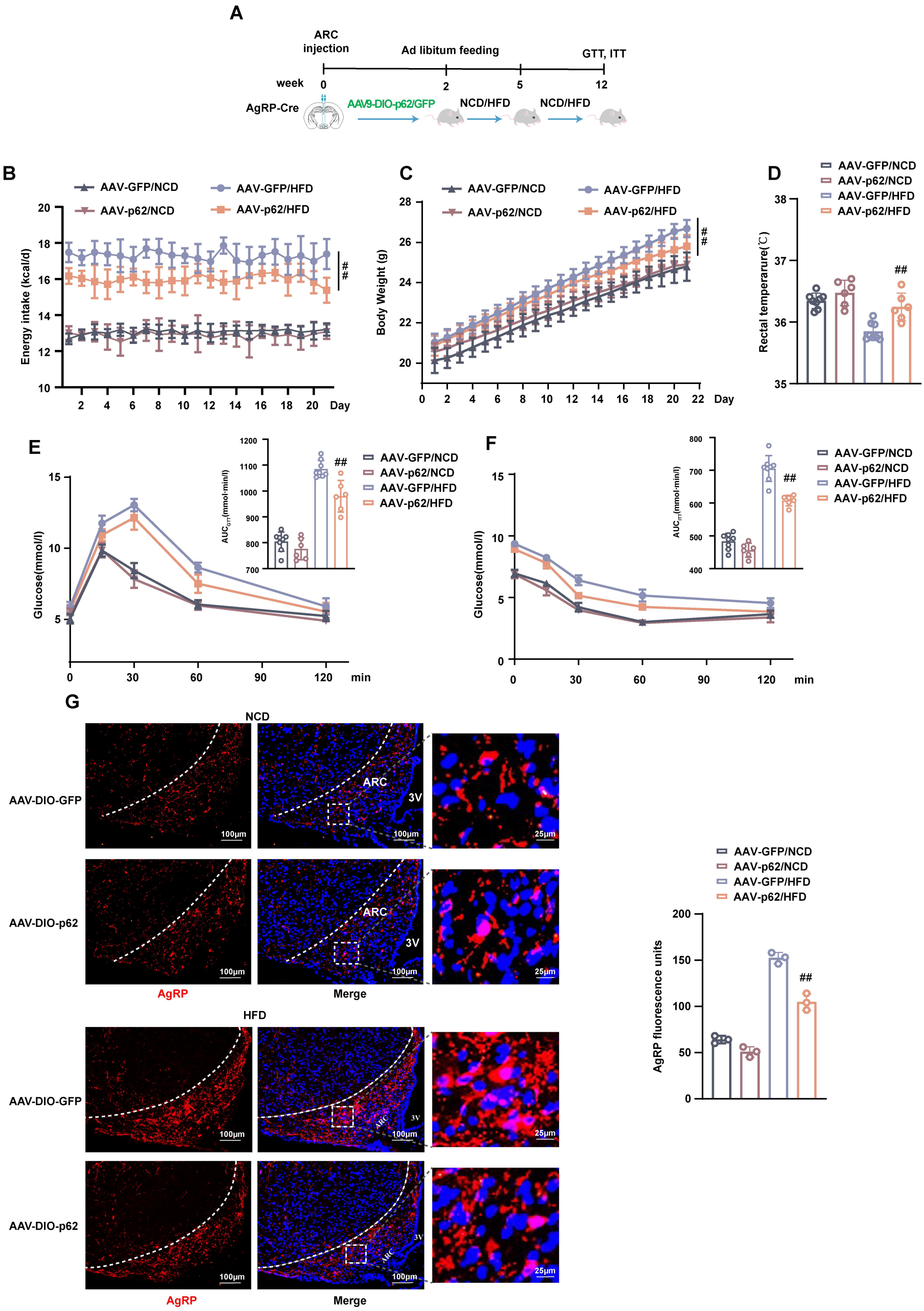

Figure S4 **Overexpression of p62 in AgRP neurons promoted energy expenditure and ameliorated glucose metabolism in HFD-fed mice. (A)** Schematic representation of the experimental procedure. Eight-week-old male AgRP-Cre mice received a bilateral ARC injection of AVV9-DIO- *p62/GFP* and were fed a NCD or HFD for 12 weeks as indicated in

the Methods. **(B)** Energy intake (n = 6-8 mice). **(C)** Body weight (n = 6-8 mice). **(D)** Rectal temperature (n = 6-8 mice). **(E and F)** Blood glucose and AUC during the GTT (E) and the ITT (F) (n = 6-8 mice). **(G)** IF staining of AgRP (left) and the RFU (right) in the ARC (n = 3 mice). NCD, normal chow diet; HFD, high-fat diet; 3V, third cerebral ventricle; ARC, arcuate nucleus; RFU, relative fluorescent units; GTT, glucose tolerance test; ITT, insulin tolerance test; AUC, the area under the curve. Data are expressed as the mean ± SD. $^{\#}p < 0.05$, $^{\#\#}p < 0.01$ *vs*. the AAV-GFP/HFD group.

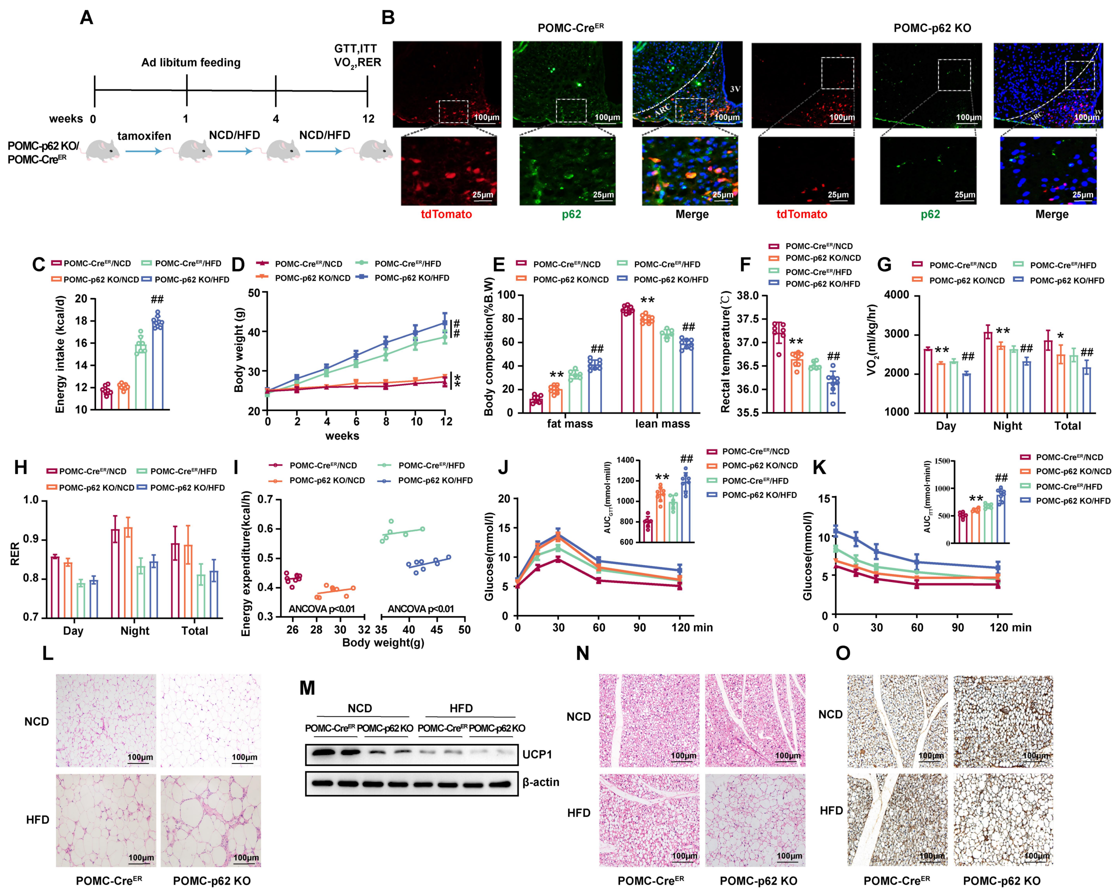

Figure S5 Inducible loss of p62 in POMC neurons reduced energy expenditure and exacerbated metabolic dysfunction. (A) Schematic representation of the experimental procedure. Eight-week-old male POMC-p62 KO and POMC-Cre^{ER} mice with tdTomato were intraperitoneally injected with tamoxifen and fed an NCD or HFD for 12 weeks as indicated in the Methods. **(B)** IF staining of POMC neurons with tdTomato (red) and p62 (green) in the ARC. **(C)** Energy intake (n = 7-9 mice). **(D)** Body weight (n = 6-8 mice). **(E)** Body composition (n = 6-8 mice). **(F)** Rectal temperature (n = 6-8 mice). **(G)** 24-h oxygen consumption (Vo_2) (n = 6-8 mice). **(H)** Respiratory exchange ratio (RER: V_{CO2}/V_{O2}) (n = 6-8 mice). **(I)** ANCOVA of the total energy expenditure versus body weight (n = 6-8 mice). **(J)** Blood glucose and AUC during the GTT (n = 6-8 mice). **(K)** Blood glucose and AUC during the ITT (n = 6-8 mice). **(L)** H&E staining in WAT (n = 3 mice). **(M)** UCP1 protein expression in WAT (n = 3 mice). **(N)** H&E staining of BAT (n = 3 mice). **(O)** UCP1 immunostaining in BAT (n = 3 mice). 3V, third cerebral ventricle; ARC, arcuate nucleus; NCD, normal chow diet; HFD, high-fat diet; GTT, glucose tolerance test; ITT, insulin tolerance test; AUC, the area under the curve; WAT, white adipose tissue; BAT, brown

adipose tissue. Data are expressed as the mean ± SD. $^{**}p < 0.01$ *vs*. POMC-Cre^{ER}/NCD; $^{\#\#}p < 0.01$ *vs*. POMC-Cre^{ER}/HFD.

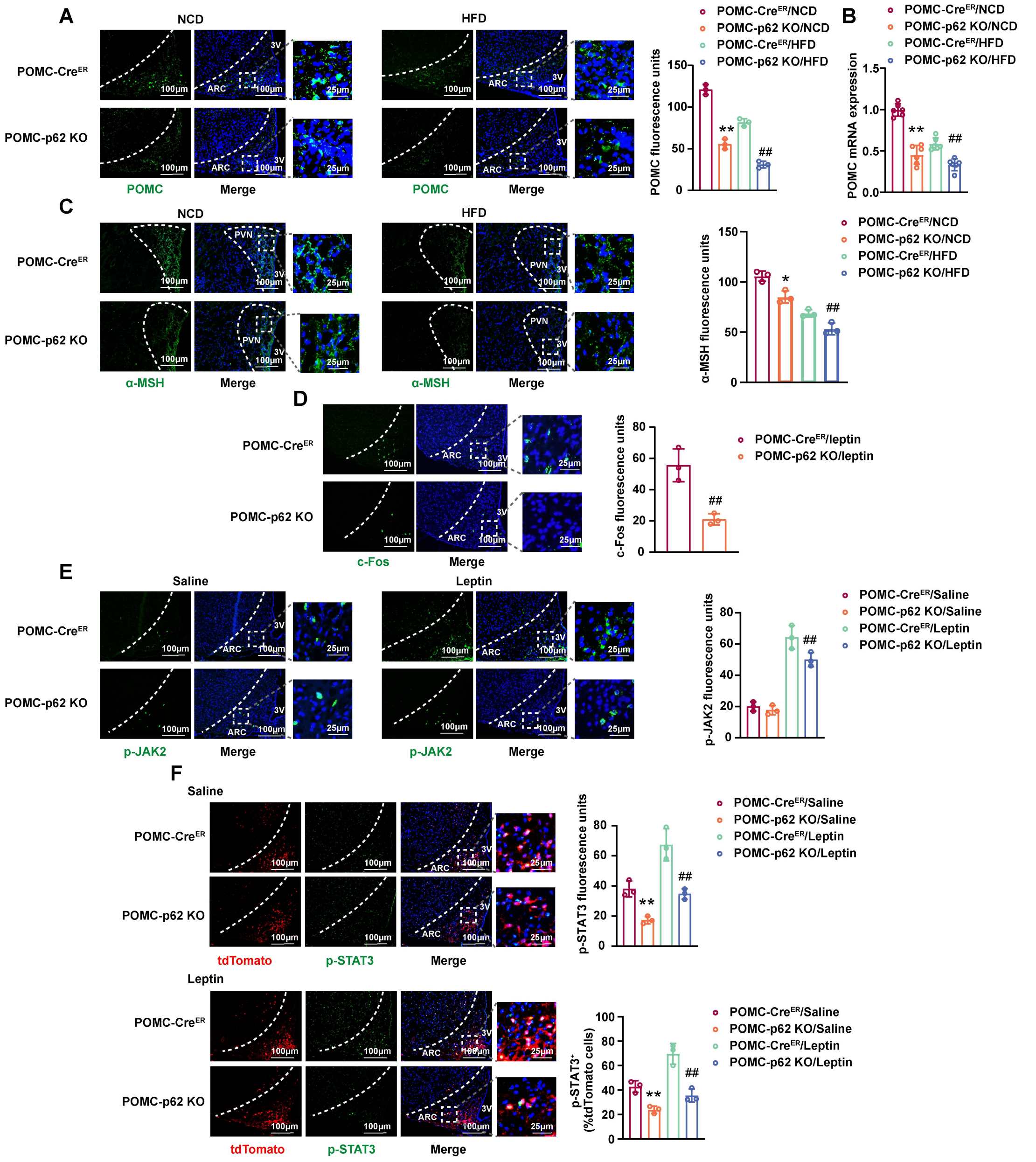

Figure S6 Inducible loss of p62 in POMC neurons inhibits leptin-JAK2/STAT3 signaling. Eight-week-old male POMC-p62 KO and POMC-Cre^{ER} mice with tdTomato were intraperitoneally injected with tamoxifen and fed an NCD or HFD for 12 weeks. Two subgroups of mice were intraperitoneally injected with leptin/saline twice a day for 3 days, as indicated in the Methods. **(A)** IF staining of POMC (left) and RFU (right) in the ARC (n = 3 mice). **(B)** POMC mRNA expression in the hypothalamus (n = 6 mice). **(C)** IF staining of α-MSH (left) and RFU (right) in the PVN (n = 3 mice). **(D)** IF staining of c-Fos (left) and the RFU (right) in the ARC (n = 3 mice). **(E)** IF staining of p-JAK2 (left) and RFU (right) in the ARC (n = 3 mice). **(F)** IF staining for phosphorylated STAT3 (left) and RFU (right) in POMC

neurons (n = 3 mice). 3V, third cerebral ventricle; ARC, arcuate nucleus; PVN, paraventricular nucleus; RFU, relative fluorescent units; NCD, normal chow diet; HFD, high-fat diet. Data are expressed as the mean ± SD. $^{**}p < 0.01$ *vs.* POMC-Cre^{ER}/NCD or POMC-Cre^{ER}/Saline; $^{\#\#}p < 0.01$ *vs*. POMC-Cre^{ER}/HFD or POMC-Cre^{ER}/Leptin.

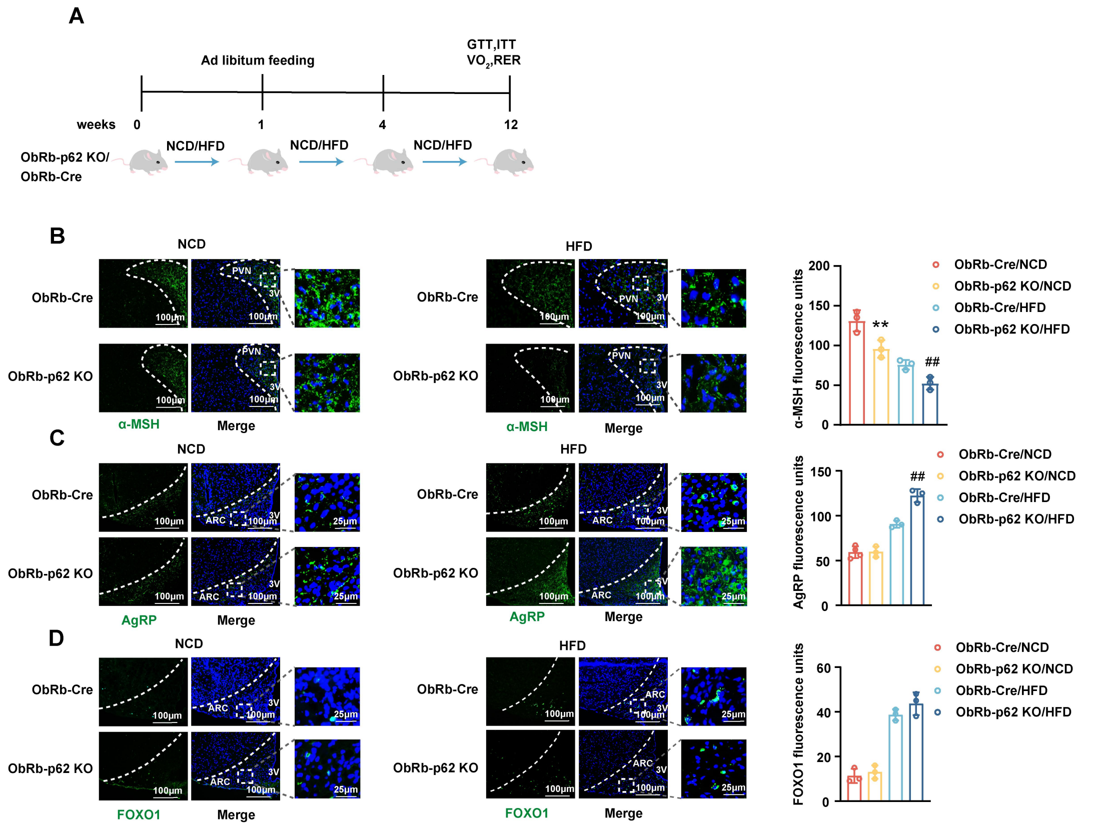

Figure S7 Effects of p62 deletion in ObRb-expressing neurons on the expression of α-MSH and AgRP. (A) Schematic representation of the experimental procedure. **(B)** IF staining of α-MSH (left) and RFU (right) in the PVN (n = 3 mice). **(C)** IF staining of AgRP (left) and RFU (right) in the ARC (n = 3 mice). **(D)** IF staining of FOXO1 (left) and RFU (right) in the ARC (n = 3 mice). 3V, third cerebral ventricle; ARC, arcuate nucleus; PVN, paraventricular nucleus; RFU, relative fluorescent units; NCD, normal chow diet; HFD, high-fat diet. Data are expressed as the mean ± SD. $^{**}p < 0.01$ *vs.* ObRb/NCD; $^{\#\#}p < 0.01$ *vs.* ObRb/HFD.

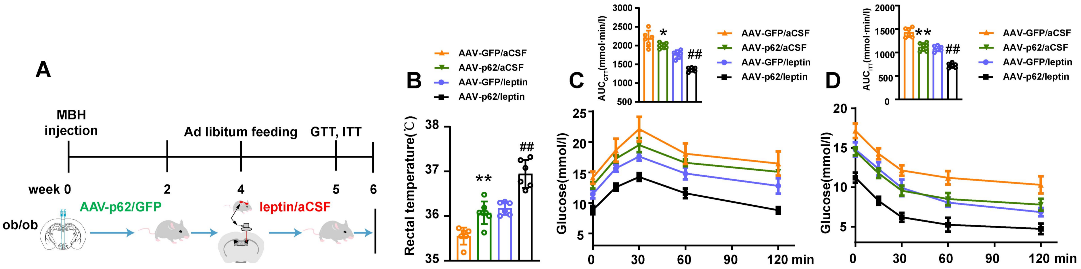

Figure S8 Overexpression of p62 in the MBH alleviates obesity induced by leptin deficiency and promotes the anti-obesity effect of leptin in ob/ob mice. (A) Schematic representation of the experimental procedure. Eight-week-old male ob/ob mice received bilateral MBH injection of AVV9-p62/GFP, followed by ICV leptin/aCSF infusion using an ALZET osmotic mini-pump for 7 days, as indicated in the Methods. **(B)** Rectal temperature (n = 6-7 mice). **(C)** Blood glucose and AUC during the GTT (n = 6-7 mice). **(D)** Blood glucose and AUC during the ITT (n = 6-7 mice). MBH, mediobasal hypothalamus; GTT, glucose tolerance test; ITT, insulin tolerance test; AUC, the area under the curve. Data are expressed as the mean ± SD. *$p < 0.05$, **$p < 0.01$ *vs*. GFP/aCSF; $^{\#\#}p < 0.01$ *vs*. GFP/Leptin.

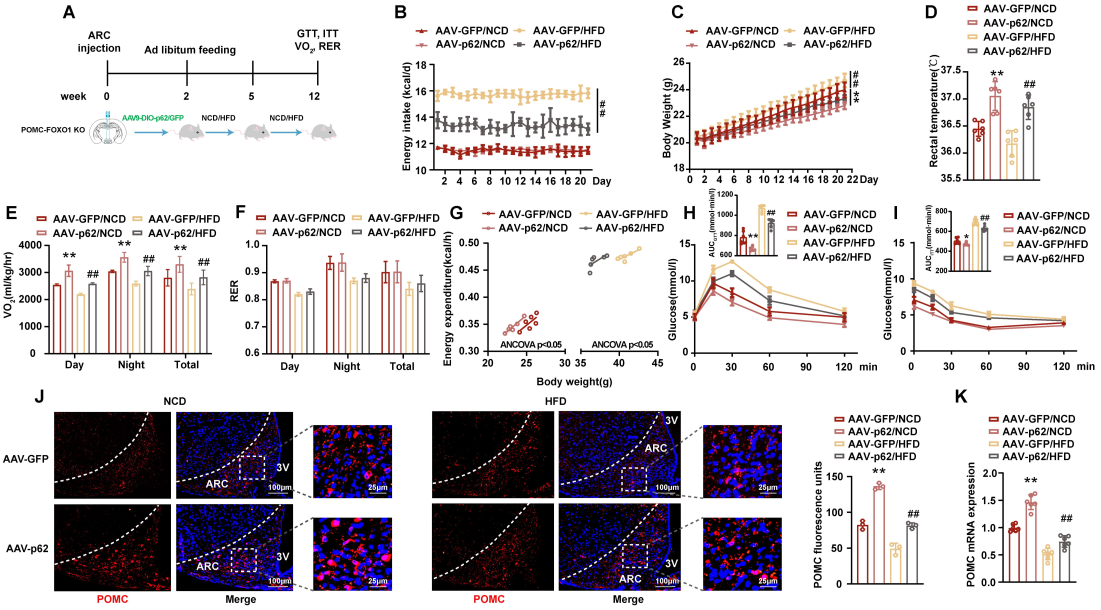

Figure S9 FoxO1 deletion in POMC neurons did not affect the function of central p62. **(A)** Schematic representation of the experimental procedure. Eight-week-old male POMC-*FoxO1* KO mice received an ARC injection of AVV9-DIO-*p62/GFP* and were fed a NCD or HFD for 12 weeks as indicated in the Methods. **(B)** Energy intake (n = 6 mice). **(C)** Body weight (n = 6 mice). **(D)** Rectal temperature (n = 6 mice). **(E)** 24-h oxygen consumption (V_{O2}) (n = 6 mice). **(F)** Respiratory exchange ratio (RER: V_{CO2}/V_{O2}) (n = 6 mice). **(G)** ANCOVA of the total energy expenditure versus body weight (n = 6 mice). **(H and I)** Blood glucose and AUC during the GTT (H) and the ITT (I) (n = 6 mice). **(J)** IF staining for POMC (left) and RFU (right) in the ARC (n = 3 mice). **(K)** POMC mRNA expression in the hypothalamus (n = 6 mice). NCD, normal chow diet; HFD, high-fat diet; 3V, third cerebral ventricle; ARC, arcuate nucleus; RFU, relative fluorescent units; GTT, glucose tolerance test; ITT, insulin tolerance test; AUC, the area under the curve. Data are expressed as the mean ± SD. **p* < 0.05, ***p* < 0.01 *vs*. GFP/NCD; ##*p* < 0.01 *vs*. GFP/HFD.
